# Synthesis-Driven Stereochemical Assignment of Marine Polycyclic Ether Natural Products

**DOI:** 10.3390/md19050257

**Published:** 2021-04-29

**Authors:** Haruhiko Fuwa

**Affiliations:** Department of Applied Chemistry, Faculty of Science and Engineering, Chuo University, 1-13-27 Kasuga, Bunkyo-ku, Tokyo 112-8551, Japan; hfuwa.50m@g.chuo-u.ac.jp

**Keywords:** polycyclic ethers, dinoflagellates, secondary metabolites, neurotoxins, total synthesis, partial synthesis, NMR spectroscopic analysis, chemical shift deviation analysis

## Abstract

Marine polycyclic ether natural products have gained significant interest from the chemical community due to their impressively huge molecular architecture and diverse biological functions. The structure assignment of this class of extraordinarily complex natural products has mainly relied on NMR spectroscopic analysis. However, NMR spectroscopic analysis has its own limitations, including configurational assignment of stereogenic centers within conformationally flexible systems. Chemical shift deviation analysis of synthetic model compounds is a reliable means to assign the relative configuration of “difficult” stereogenic centers. The complete configurational assignment must be ultimately established through total synthesis. The aim of this review is to summarize the indispensable role of organic synthesis in stereochemical assignment of marine polycyclic ethers.

## 1. Introduction

**Figure 1:**
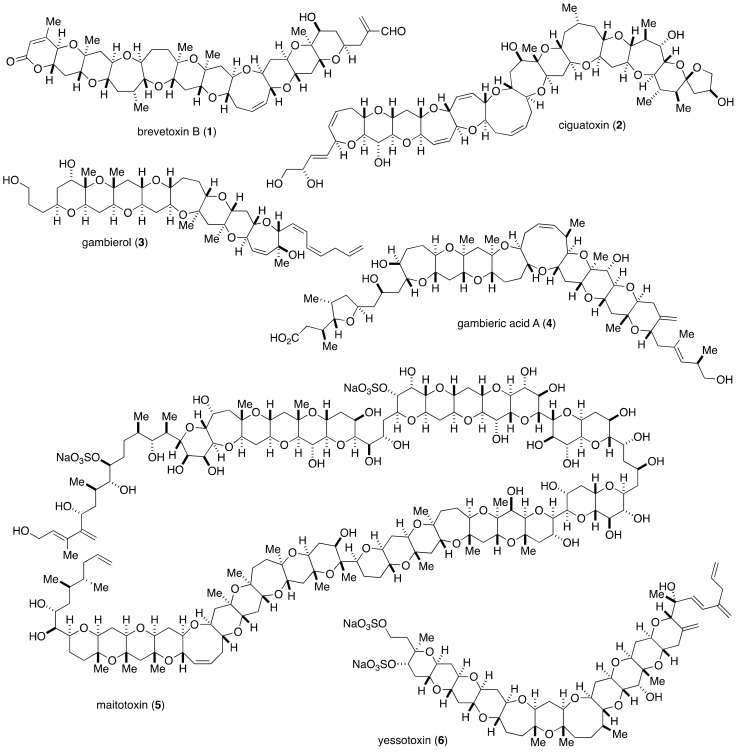
Structures of selected marine polycyclic ether natural products.

Marine polycyclic ethers, produced as secondary metabolites by marine microalgae, mainly dinoflagellates, have gained significant interest from the chemical community because of their impressively huge molecular architecture and diverse biological functions (Figure 1) [1,2,3]. The majority of this family of natural products were identified as marine toxins responsible for human intoxications and massive fish kills. At the molecular level, many of marine polycyclic ethers are known to interact specifically with ion channels either as agonists, antagonists or partial agonists. Thus, marine polycyclic ethers are pharmacologically useful probes for structural and functional analyses of ion channels [4,5,6,7,8]. In this context, it is obvious that the structure determination of marine polycyclic ethers is of utmost importance to precisely discuss their structure–activity relationship as well as mode of interaction with ion channels.

The first member of this family of natural products to be structurally elucidated was brevetoxin B (**1**). The structure of brevetoxin B was established by Nakanishi and coworkers through an X-ray crystallographic analysis [9]. Thereafter, a number of marine polycyclic ethers have been isolated and structurally characterized. The common structural motif shared among this family of natural products is the ladder-shaped polycyclic ether skeleton fused in a *trans*/*syn*/*trans* fashion, and the ring size of cyclic ethers ranges from five to nine membered. A plausible biosynthetic mechanism postulated by Nakanishi/Shimizu involves a cascade cyclization of polyepoxides consisting of a single carbon chain to “zip up” the fused polycyclic ether skeleton [10,11,12,13]. Vilotijevic and Jamison demonstrated a polyepoxide cyclization cascade in neutral H_2_O at 70 °C to produce *trans*-fused polytetrahydropyrans, thereby supporting the Nakanishi’s biosynthetic hypothesis [14].

The extraordinarily complex structure of marine polycyclic ethers has been characterized mostly by NMR spectroscopic analysis due to their extremely limited availability from natural sources as well as their physicochemical properties unsuitable for X-ray crystallographic analysis. From the late 1980s, the structure determination of ciguatoxins [15,16,17], gambieric acids [18,19,20], gambierol [21,22], maitotoxin [23,24,25,26,27], and yessotoxins [28,29,30] was accomplished by the Yasumoto group. Yasumoto and coworkers relied heavily on 2D-NMR and in certain cases 3D-NMR analyses for determining the gross structure and the relative configuration of the polycyclic ether skeleton of these compounds. However, NMR spectroscopic analysis has its own limitations, including configurational assignment of stereogenic centers within conformationally flexible systems.

Stereocontrolled synthesis enables preparation of a series of diastereomeric compounds of a natural product in question. Comparison of the NMR spectroscopic data of diastereomeric compounds with those of the parent natural product (chemical shift deviation analysis) is a powerful means to elucidate the configuration of difficult-to-assign stereogenic centers [31,32,33,34,35]. However, not all the possible diastereomers need to be prepared; the number of possible diastereomers can be reduced through conformational analysis of the parent natural product. Synthesis and NMR analysis of a single model compound may be sufficient to confirm the configuration of a most likely stereoisomer. In any case, it should be emphasized that the conformation of a model compound with correct configuration must reproduce that of the corresponding moiety of the parent natural product, and that the complete configurational assignment must be ultimately established through stereocontrolled total synthesis. This review summarizes selected examples of synthesis-driven stereochemical assignment of marine polycyclic ethers to highlight the significance of organic synthesis in structure determination of natural products.

## 2. Maitotoxin

Maitotoxin (**5**) was first isolated from the surgeonfish *Ctenochaetus striatus* as one of the causative toxins of ciguatera seafood poisoning [36] and was subsequently identified to originate from the dinoflagellate *Gambierdiscus toxicus* [37]. This natural product is the largest non-biopolymer secondary metabolite known to date and exhibits diverse biological activities that are supposed to be triggered by intracellular Ca^2+^ elevation [37,38,39,40,41,42]. The LD_50_ value of maitotoxin against mice (ddY strain, 16–20 g body weight) was determined to be 50 ng/kg (intraperitoneal, i.p.) [25]. The gross structure of maitotoxin, which consists of 142 carbon atoms and 32 cyclic ethers ranging from six to eight membered, was determined by chemical degradation of the authentic sample and extensive 2D- and 3D-NMR and negative FAB MS/MS experiments due to extremely heavy overlapping of the ^1^H and ^13^C NMR signals [23,24,25]. The relative configuration of the fused polycyclic ether domains was assigned by Murata, Yasumoto and coworkers on the basis of NOESY correlations and ^3^*J*_H,H_ values, with the aid of molecular mechanics calculations. However, the relative configuration of the remaining acyclic portions and the absolute configuration of maitotoxin could not be assigned solely by NMR spectroscopic analysis.

### 2.1. Determination of the Absolute Configuration of Maitotoxin by the Tachibana Group and Yasumoto Group

The configurational assignment of the stereogenic centers included in the acyclic portions and the determination of the absolute configuration of maitotoxin were achieved by a collaborative work of the Tachibana group and the Yasumoto group through extensive NMR spectroscopic analysis on natural and ^13^C-enriched maitotoxin as well as synthesis of model compounds and comparison with those of the natural product [26,27,43,44,45,46].

#### 2.1.1. Stereochemical Assignment of the C63–C68 Linkage

**Figure 2:**
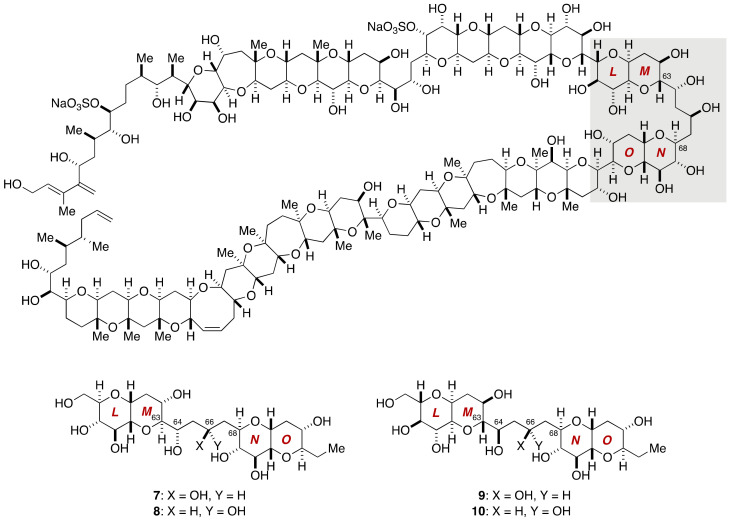
Candidate diastereomers **7**–**10** for stereochemical assignment of the C63–C68 linkage of maitotoxin.

The C63–C68 linkage is the acyclic portion flanked with the LM- and NO-rings and contains two stereogenic centers. Detailed conformational analysis on the basis of NOEs and ^3^*J*_H,H_ values led the Tachibana group to synthesize four candidate diastereomers **7**–**10** (Figure 2) for this partial structure [43,44].

**Figure 3:**
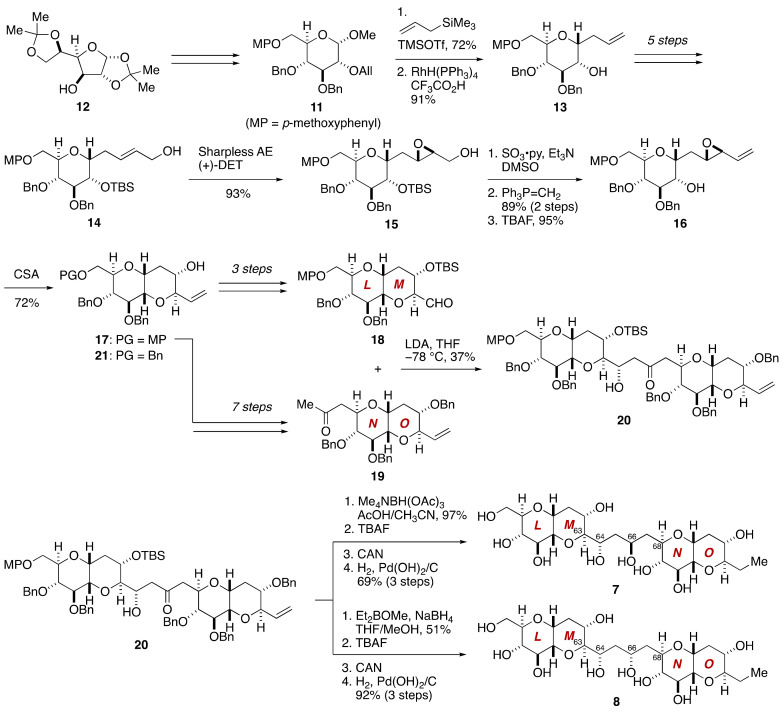
Synthesis of candidate diastereomers **7** and **8** for stereochemical assignment of the C63–C68 linkage.

The synthesis of model compounds **7** and **8** started with methyl glycoside **11**, which was prepared from 1,2,5,6-diisopropylidene-α-d-glucose (**12**) (Figure 3). Allylation of **11** with allyltrimethylsilane in the presence of TMSOTf [47], followed by cleavage of the allyl ether, gave alcohol **13**. After a five-step sequence of manipulations, Sharpless asymmetric epoxidation [48] of the resultant allylic alcohol **14** provided epoxy alcohol **15**. Oxidation, Wittig methylenation, and desilylation gave rise to vinyl epoxide **16**, whose treatment with CSA triggered 6-*endo* cyclization [49] to afford alcohol **17**. This compound served as a common precursor for the LM- and NO-ring fragments **18** and **19**. Aldol coupling of **18** and **19** using LDA as a base provided β-hydroxy ketone **20** in 37% yield as the major diastereomer. The configuration of the C64 stereogenic center of **20** was confirmed by derivatization. Evans 1,3-*anti* reduction [50] of **20** followed by a three-step global deprotection of the protecting groups furnished model compound **7**. Meanwhile, Narasaka–Prasad 1,3-*syn* reduction [51] of **20** and subsequent three-step deprotection led to model compound **8**. The stereochemical consequence of the Narasaka–Prasad 1,3-*syn* reduction of **20** was confirmed by derivatization of the product 1,3-diol to the corresponding acetonide. The remaining model compounds **9** and **10** were similarly prepared starting from *ent*-**21** and **19**. Detailed comparison of the ^1^H and ^13^C NMR spectroscopic data of model compounds **7**–**10**, recorded in CD_3_CN/H_2_O (1:1), with those of the corresponding part of the authentic maitotoxin clearly indicated that **7** represents the relative configuration of the C63–C68 acyclic linkage.

#### 2.1.2. Stereochemical Assignment of the C35–C39 Acyclic Linkage

**Figure 4:**
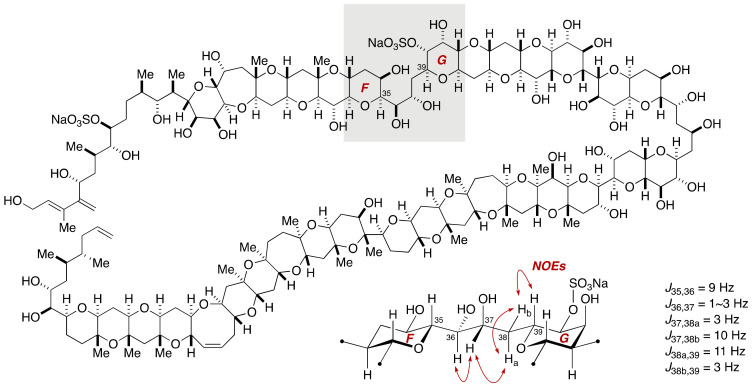
Deduced configuration and conformation of the C35–C39 linkage of maitotoxin. Double-ended arrows denote NOEs.

**Figure 5:**
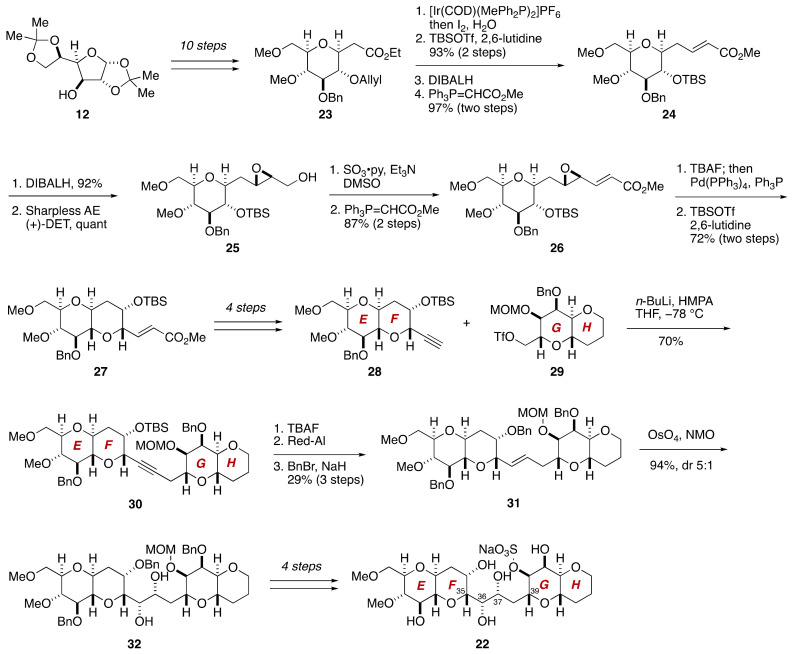
Synthesis of model compound **22** for stereochemical assignment of the C35–C39 linkage.

The C35–C39 acyclic linkage connects the F- and G-rings and contains two stereogenic centers. Based on the NOESY and E.COSY spectra of maitotoxin, the Tachibana group deduced the relative configuration and the conformation of this acyclic moiety as shown in Figure 4. To confirm this assignment, model compound **22** that represents the EF/GH-ring system was synthesized from ester **23**, which was prepared from 1,2,5,6-diisopropylidene-α-d-glucose (**12**) in ten steps [45] (Figure 5).

After two-step protecting group manipulations on **23**, DIBALH reduction and Wittig olefination gave α,β-unsaturated ester **24**. DIBALH reduction of **24** followed by Sharpless asymmetric epoxidation provided epoxy alcohol **25**. Oxidation and Wittig olefination led to α,β-unsaturated ester **26**, which upon exposure to TBAF and then to Pd(PPh_3_)/Ph_3_P induced 6-*endo* cyclization [52], affording bicyclic ether **27**, after silylation of the cyclization product. Subsequent four-step manipulations gave rise to the EF-ring alkyne **28**. The requisite coupling partner, the GH-ring triflate **29**, was synthesized from methyl α-d-glucopyranoside by routine chemistry [45]. Coupling of the EF-ring alkyne **28** and the GH-ring triflate **29** afforded coupling product **30** in 70% yield. After desilylation with TBAF, the resultant alkyne was stereospecifically reduced with Red-Al^®^ to give, after benzylation, (*E*)-olefin **31**. Stereoselective dihydroxylation of **31** under standard catalytic conditions provided diol **32** in 94% yield with approximately 5:1 diastereoselectivity, in accordance with Kishi’s empirical stereochemical model [53]. The stereochemical outcome of the dihydroxylation was later confirmed on the basis of *J*_C,H_ and *J*_H,H_ values [46,54]. Subsequent four-step manipulations including sulfonylation of the C40 hydroxy group furnished model compound **22**. The ^1^H and ^13^C NMR spectroscopic data of the C35–C39 acyclic linkage of **22**, collected in C_5_D_5_N/CD_3_OD (1:1), were in excellent agreement with those of the corresponding part of maitotoxin, thereby confirming the configurational assignment of the C35–C39 acyclic linkage.

#### 2.1.3. Stereochemical Assignment of the C1–C14 Side Chain

**Figure 6:**
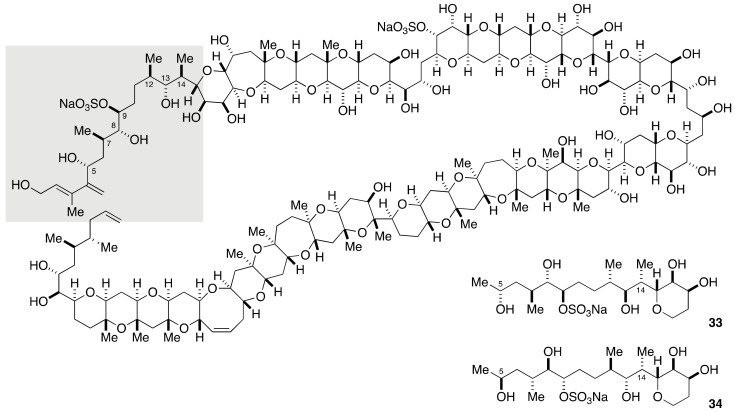
Two diastereomeric model compounds **33** and **34** for stereochemical assignment of the C1–C14 side chain.

The C1–C14 side chain of maitotoxin involves seven stereogenic centers, and 2^7^ = 128 stereoisomers are possible for this acyclic portion (Figure 6). Obviously, however, the synthesis of all the possible stereoisomers is unrealistic. To reduce the number of candidate diastereomers, the Tachibana/Yasumoto group assigned all but one relative configurations of the stereogenic centers within the C1–C14 side chain by means of extensive conformational analysis. Since NOE-based conformational analysis was unsuitable for the conformationally flexible C5–C9 moiety, the configuration of the C5, C7, C8, and C9 positions was deduced based on *J*-based configurational analysis (JBCA) [54]. The JBCA method is widely applicable to conformationally non-biased acyclic compounds and involves measurement of long-range carbon-proton (^2,3^*J*_C,H_) and proton-proton (^3^*J*_H,H_) coupling constants. The C9–C12 moiety, on the other hand, appeared relatively rigid in conformation and the relative configuration between C9 and C12 could be correlated through conformational analysis based on NOEs and ^3^*J*_H,H_ values. The C12–C14 moiety was suggested to exist as an equilibrating mixture of two conformers, and the relative configurations of C12/C13 and C14/C15 were assigned by exploiting the JBCA method. Because the relative configuration between C13 and C14 could not be assigned by NMR spectroscopic analysis, the Tachibana/Yasumoto group determined to synthesize two diastereomeric model compounds **33** and **34** to compare their NMR spectroscopic data with those of the corresponding part of maitotoxin.

**Figure 7:**
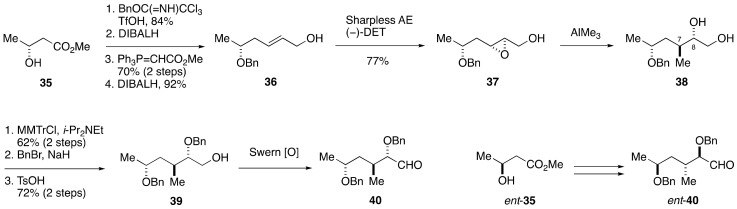
Synthesis of the left half aldehydes **40** and *ent*-**40**.

The synthesis of the left half of compound **33** started with methyl (*R*)-3-hydroxybutyrate (**35**), as shown in Figure 7. A four-step sequence of standard manipulations gave allylic alcohol **36**. Sharpless asymmetric epoxidation delivered epoxy alcohol **37**, which upon treatment with AlMe_3_ [55,56] underwent regioselective and stereospecific epoxide opening to afford 1,2-diol **38** with desired configuration at the C7 and C8 positions. Subsequent three-step protecting group manipulations gave alcohol **39**, which was oxidized under Swern conditions [57] to provide aldehyde **40**. The enantiomer of **40**, i.e., *ent*-**40**, was prepared in a similar fashion starting from *ent*-**35**.

**Figure 8:**
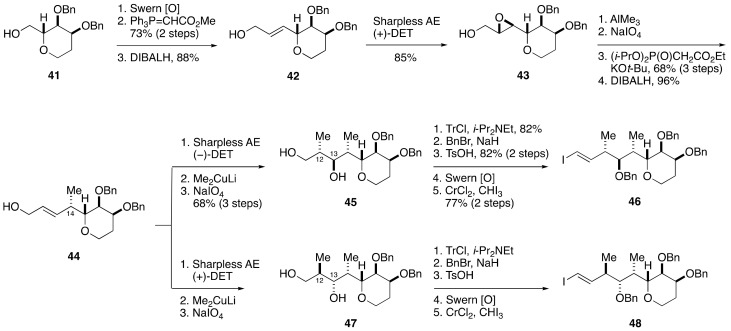
Synthesis of the right half iodoolefins **46** and **48**.

The synthesis of the right half of compounds **33** and **34** commenced with Swern oxidation of alcohol **41**, readily prepared from tri-*O*-acetyl-d-glucal in 10 steps, followed by Wittig reaction of the derived aldehyde (Figure 8). DIBALH reduction of the resultant α,β-unsaturated ester gave allylic alcohol **42**. Sharpless asymmetric epoxidation of **42** delivered epoxy alcohol **43**. Regio- and stereospecific methylation of **43** was followed by cleavage of the resultant 1,2-diol with NaIO_4_.

The subsequent Horner–Wadsworth–Emmons reaction and DIBALH reduction led to allylic alcohol **44** with correct configuration at the C14 stereogenic center. Sharpless asymmetric epoxidation of **44** using (−)-DET/Ti(O*i*-Pr)_4_ as a chiral catalyst afforded an epoxy alcohol, whose exposure to Me_2_CuLi opened the epoxide in a regioselective/stereospecific manner [58] to provide 1,3-diol **45** after periodate workup, and thereby successfully installed the requisite C12 and C13 stereogenic centers. After three-step protecting group manipulations, the derived alcohol was oxidized and then iodoolefinated [59] to furnish iodoolefin **46**. The diastereomeric iodoolefin **48** was synthesized from allylic alcohol **44** in the same way as **46**, except for the Sharpless asymmetric epoxidation, where (+)-DET/Ti(O*i*-Pr)_4_ was used as a chiral catalyst to create the C12 and C13 stereogenic centers.

**Figure 9:**
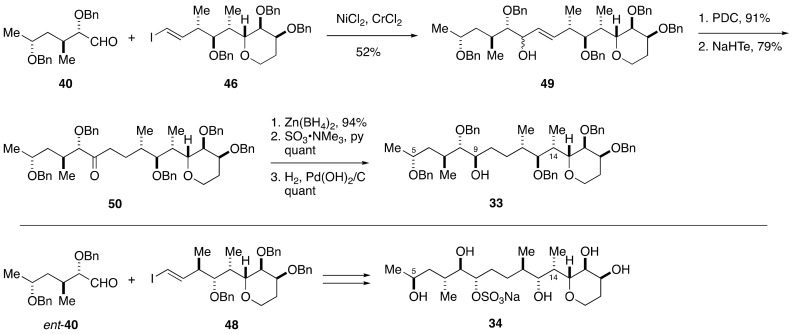
Synthesis of model compounds **33** and **34**.

The Nozaki–Hiyama–Kishi reaction [60,61] of aldehyde **40** and iodoolefin **46** afforded allylic alcohol **49** in 52% yield (Figure 9). After oxidation, the resultant α,β-unsaturated ketone was reduced with NaHTe [62] to give ketone **50**. Chelate-controlled reduction of **50** with Zn(BH_4_)_2_ [63] provided an alcohol with desired configuration at C9. Sulfonylation and hydrogenolysis of the benzyl ethers afforded compound **33**. Following a similar synthetic strategy, compound **34** was synthesized from *ent*-**40** and iodoolefin **48**. Comparison of the ^1^H and ^13^C NMR spectroscopic data of **33** and **34** with those of the corresponding moiety of natural maitotoxin showed that **33** was in good accordance with the natural product with respect to the ^13^C NMR chemical shift data. The ^3^*J*_H,H_ values of the C7–C9 and C12–C15 portions of **33** were also in good agreement with those of natural maitotoxin. From these results, the relative configuration of the C1–C14 side chain was successfully determined.

#### 2.1.4. Stereochemical Assignment of the C135–C142 Side Chain and Absolute Configuration of Maitotoxin

**Figure 10:**
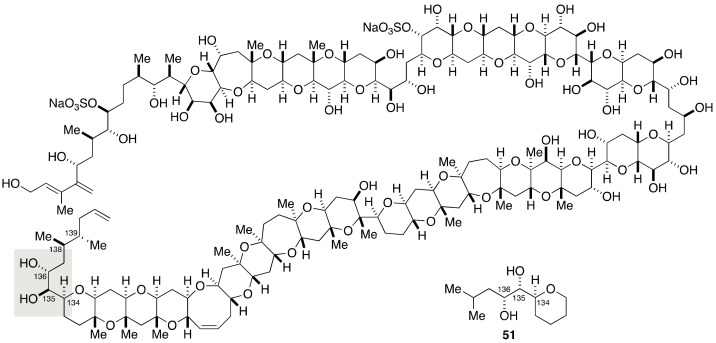
Model compound **51** for stereochemical assignment of the C134–C136 portion.

Among the four stereogenic centers included in the C135–C142 side chain of maitotoxin, the relative configuration between C136 and C138 positions could be unambiguously assigned through a *J*-based configurational analysis (Figure 10). The relative configuration of the C134, C135, and C136 stereotriad was also assignable by means of a JBCA. However, the NOEs and ^3^*J*_H,H_ values observed for this portion suggested the presence of two interconverting conformers. To exclude the ambiguity in the assignment made by the JBCA method, the Tachibana/Yasumoto group synthesized model compound **51**.

**Figure 11:**
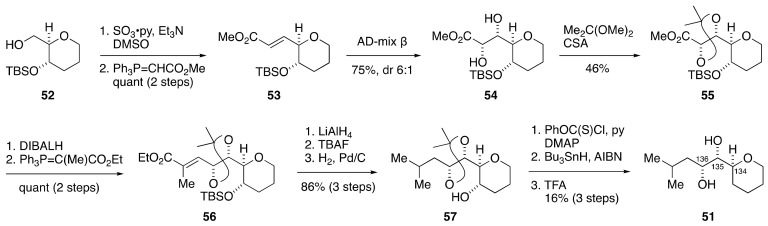
Synthesis of model compound **51**.

The synthesis of model compound **51** started from known alcohol **52**, which was available in five steps from tri-*O*-acetyl-d-glucal [64] (Figure 11). Oxidation followed by Wittig olefination gave α,β-unsaturated ester **53**. Sharpless asymmetric dihydroxylation [65] of **53** with AD-mix β led to diol **54** with 6:1 diastereomer ratio. After protection of **54** as its acetonide, the desired diastereomer **55** was isolated in a stereochemically pure form by column chromatography using silica gel. DIBALH reduction and Wittig reaction delivered α,β-unsaturated ester **56**, which was converted to alcohol **57** via a three-step sequence of manipulations. Deoxygenation of **57** through tin hydride reduction of a thionocarbonate [66], followed by removal of the acetonide under acidic conditions, afforded model compound **51**. The ^2^*J*_C,H_ and ^3^*J*_H,H_ values of the C134–C136 portion of **51** corresponded with those of natural maitotoxin, thereby confirming the configurational assignment by the JBCA method.

**Figure 12:**
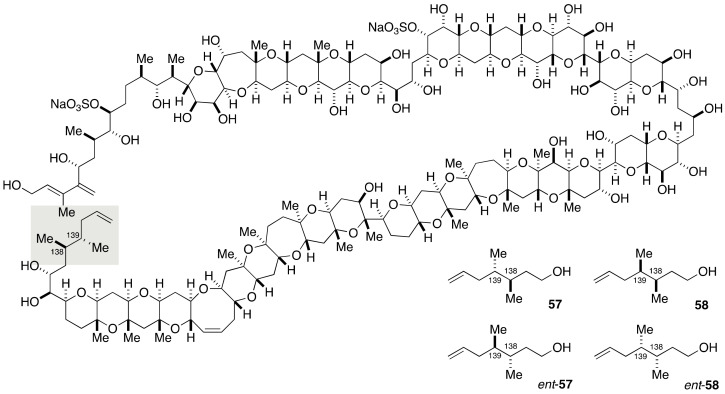
Model compounds **57**, *ent*-**57**, **58**, and *ent*-**58** for stereochemical assignment of the C138–C139 portion and determination of the absolute configuration.

The configurational assignment of the C139 stereogenic center was not possible by the JBCA method because the broadened signals of H-138 and H-139 precluded the measurement of ^3^*J*_C,H_ and ^3^*J*_H,H_ values [27]. The Tachibana/Yasumoto group synthesized four stereoisomeric model compounds **57**, *ent*-**57**, **58**, and *ent*-**58** to establish the relative configuration between C138 and C139 positions and at the same time the absolute configuration by comparing these stereoisomeric model compounds with the authentic degradation material (Figure 12).

**Figure 13:**
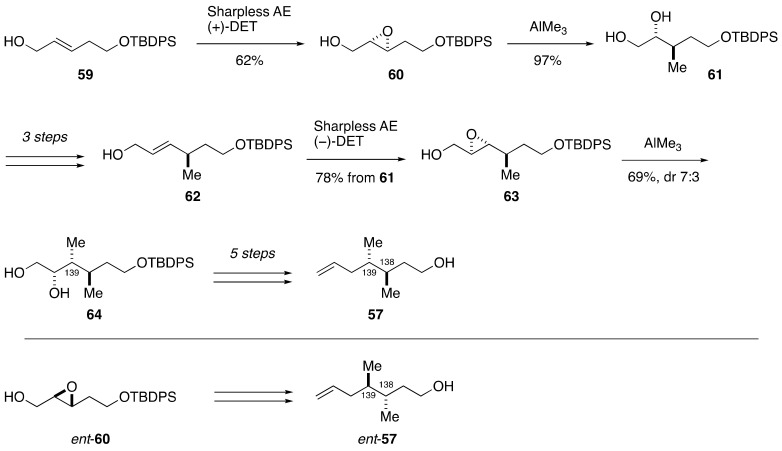
Synthesis of model compounds **57** and *ent*-**57**.

The synthesis of model compound **57** commenced with Sharpless asymmetric epoxidation of allylic alcohol **59** using (+)-DET/Ti(O*i*-Pr)_4_ as a chiral catalyst to give epoxy alcohol **60** (Figure 13). Regioselective and stereospecific methylation of **60** with AlMe_3_ delivered 1,2-diol **61**. After a three-step sequence of manipulations, Sharpless asymmetric epoxidation of allylic alcohol **62** using (−)-DET/Ti(O*i*-Pr)_4_ as a chiral catalyst provided epoxy alcohol **63**. Treatment of **63** with AlMe_3_ gave 1,2-diol **64** as a 7:3 mixture of diastereomers at the C139 stereogenic center. The stereochemical consequence of the methylation was unexpected but the major diastereomer **64** was isolated by column chromatography using silica gel and carried forward. A five-step sequence of manipulations afforded model compound **57**. The enantiomer of **57**, i.e., *ent*-**57**, was prepared in a similar manner.

**Figure 14:**
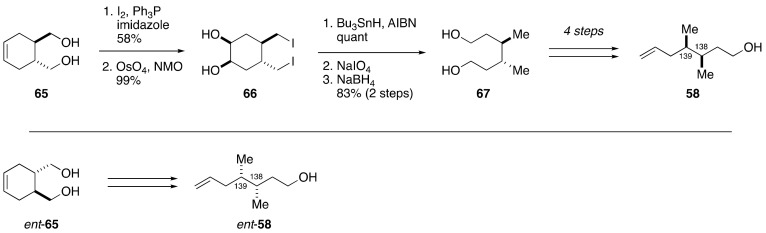
Synthesis of model compounds **58** and *ent*-**58**.

The synthesis of model compound **58** started from diol **65**, which was prepared in two steps from di-(+)-menthyl fumarate via a Diels-Alder reaction with 1,3-butadine and a subsequent reduction using LiAlH_4_ (Figure 14) [67,68]. Iodination of **65** followed by dihydroxylation gave 1,2-diol **66**. Deiodination under tin hydride conditions, periodate cleavage, and NaBH_4_ reduction delivered diol **67**. A four-step sequence of desymmetrization furnished model compound **58**. The independent synthesis of **58** confirmed the relative configuration of **57**. The synthesis of *ent*-**58** was carried out in a similar manner as that described for **58**.

Chiral GC/MS analysis of four stereoisomeric compounds **57**, *ent*-**57**, **58**, and *ent*-**58** with authentic reference prepared via degradation of natural maitotoxin (NaIO_4_ oxidation to cleave the C135/C136 *vic*-diol moiety, followed by NaBH_4_ reduction of the derived aldehydes) clearly demonstrated that the retention time and mass spectrum of **57** matched those of the authentic degradation sample. Accordingly, the absolute configuration of maitotoxin was determined to be shown by structure **5** [27].

### 2.2. Determination of the Relative Configuration of Maitotoxin by the Kishi Group

The Kishi group also elucidated, independently from the Tachibana/Yasumoto group, the complete relative configuration of maitotoxin [69]. Kishi et al. assigned the four acyclic portions of maitotoxin, i.e., the C1–C15, C35–C39, C63–C68, and C134–C142 portions, on the basis of stereocontrolled synthesis and NMR spectroscopic analysis of model compounds.

#### 2.2.1. Stereochemical Assignment of the C1–C15 Side Chain

**Figure 15:**
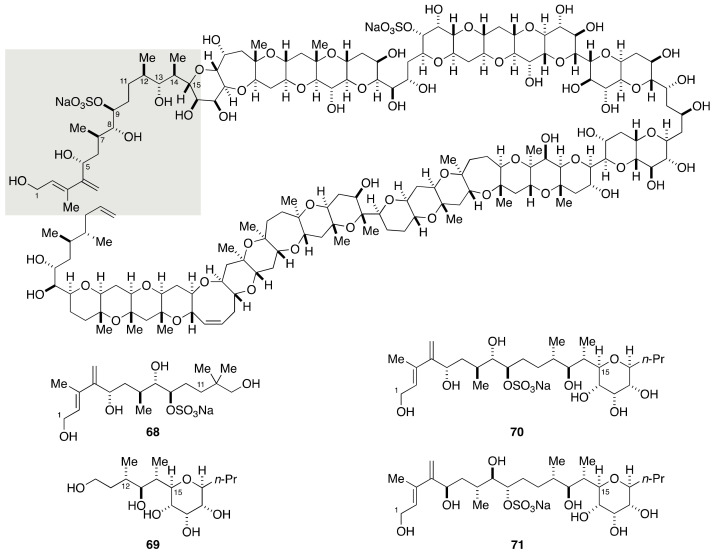
Model compounds **68**–**71** for stereochemical assignment of the C1–C15 side chain.

As described above, 128 stereoisomers are possible for the C1–C15 side chain. To narrow down the number of candidate diastereomers, Kishi divided this portion into the C1–C11 and C11–C15 portions and assigned the relative configuration of these two portions individually (Figure 15). This approach was based on their previous work on the stereochemical assignment of AAL toxin T_A_ and fumonisin B_2_ backbone [70,71,72], and subsequently extended to the concept of “universal NMR database” [73,74,75]. In the present case, two methylene groups (C10 and C11) separate two stereoclusters, i.e., C5–C9 and C12–C15, and the steric and/or stereoelectronic interactions between these two stereoclusters were assumed to be very small. The Kishi group synthesized eight possible diastereomers for the C5–C9 portion and found **68** to be in accordance with the corresponding portion of natural maitotoxin with respect to ^1^H and ^13^C NMR data. The relative configuration of the C11–C15 portion was similarly assigned as shown by structure **69** through the synthesis of eight possible diastereomers.

**Figure 16:**
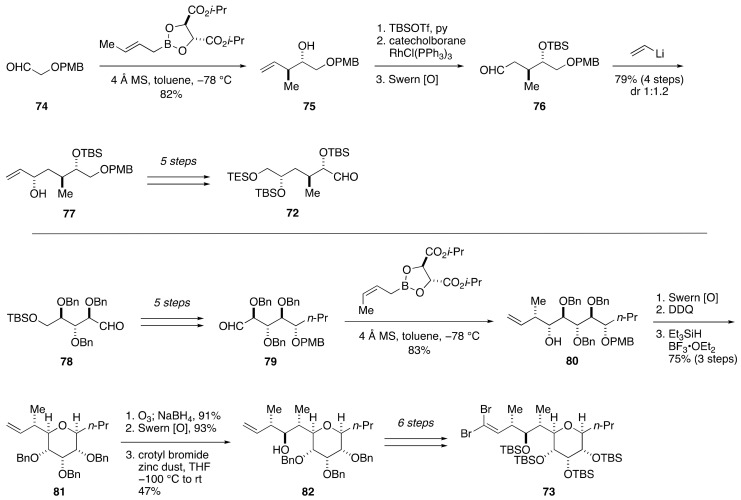
Syntheses of aldehyde **72** and dibromoolefin **73**.

**Figure 17:**
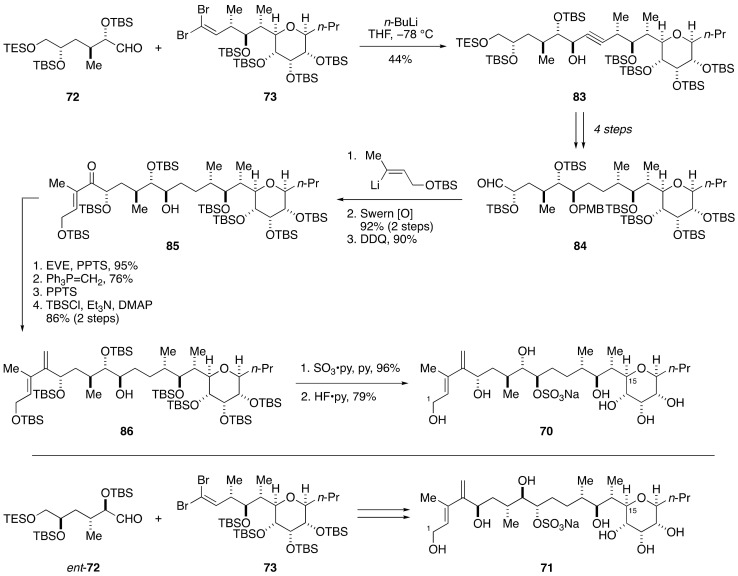
Synthesis of model compounds **70** and **71** for stereochemical assignment of the C1–C15 side chain.

Based on these results, Kishi et al. determined to synthesize two candidate diastereomers **70** and **71** for the assignment of the relative configuration of the C1–C15 side chain. The synthesis of **70** was achieved in a convergent manner through a coupling of aldehyde **72** and dibromoolefin **73** (Figure 16 and Figure 17). The synthesis of **72** started with Roush asymmetric crotylation [76] of (*p*-methoxybenzyloxy)acetaldehyde (**74**) to give homoallylic alcohol **75**, which was converted to aldehyde **76** via a three-step sequence of manipulations (Figure 16). Addition of vinyllithium to **76** provided allylic alcohol **77** as an approximately 1:1 mixture of diastereomers, which were separable by column chromatography using silica gel. A subsequent five-step sequence of standard manipulations led to aldehyde **72**. Meanwhile, the synthesis of **73** started from known aldehyde **78**, which was available from d-ribose [77]. A five-step sequence of manipulations led to aldehyde **79**, which was reacted with Roush’s (*Z*)-(*R*,*R*)-crotylboronate to give homoallylic alcohol **80** exclusively. Swern oxidation, removal of the PMB group, and reductive etherification afforded tetrahydropyran **81**. After cleavage of the double bond, the resultant aldehyde was crotylated using crotyl bromide and zinc dust to give homoallylic alcohol **82**. This compound was elaborated to dibromoolefin **73** through a six-step sequence of manipulations.

Coupling of aldehyde **72** and dibromoolefin **73** was efficiently achieved by treatment of **73** with *n*-BuLi, followed by addition of the resultant lithium acetylide to **72**, to provide propargylic alcohol **83** in 97% yield, albeit with moderate diastereoselectivity (Figure 17). The desired isomer was isolated by silica gel column chromatography in 44% yield. After four steps of manipulations including the reduction of the alkyne and selective liberation/oxidation of the primary alcohol, addition of ((*E*)-4-*t*-butyldimethylsilyloxy)-2-buten-2-yllithium to the derived aldehyde **84**, followed by oxidation of the resultant allylic alcohol and deprotection of the PMB group, delivered ketone **85**. After masking the hydroxy group of **85** as its EE ether, Wittig methylenation of the carbonyl group and ensuing two-step protecting group manipulations gave alcohol **86**. Sulfonylation and global deprotection of the silyl groups furnished model compound **70**. In a similar fashion, model compound **71** was synthesized from *ent*-**72** and **73**. Comparison of the ^1^H and ^13^C NMR spectroscopic data of **70** and **71** with those of the corresponding part of natural maitotoxin enabled assignment of the relative configuration of the C1–C15 side chain to be shown by structure **70** [69].

#### 2.2.2. Stereochemical Assignment of the C35–C39 Acyclic Portion

**Figure 18:**
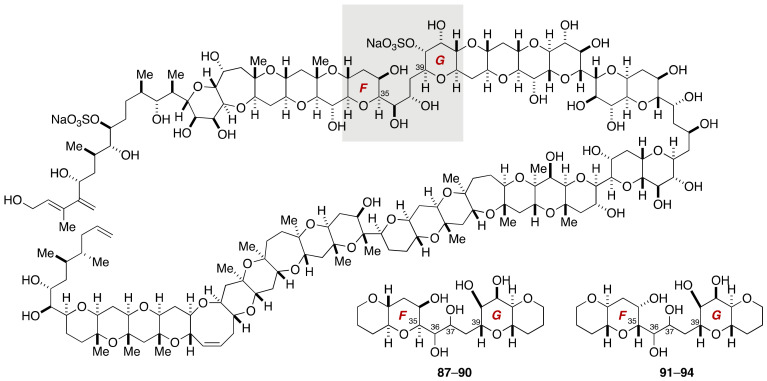
Model compounds **87**–**94** for stereochemical assignment of the C35–C39 side chain.

Kishi et al. synthesized all the possible diastereomers of the EF/GH-ring system (Figure 18, **87**–**94**) to elucidate the relative configuration of the C35–C39 acyclic portion.

**Figure 19:**
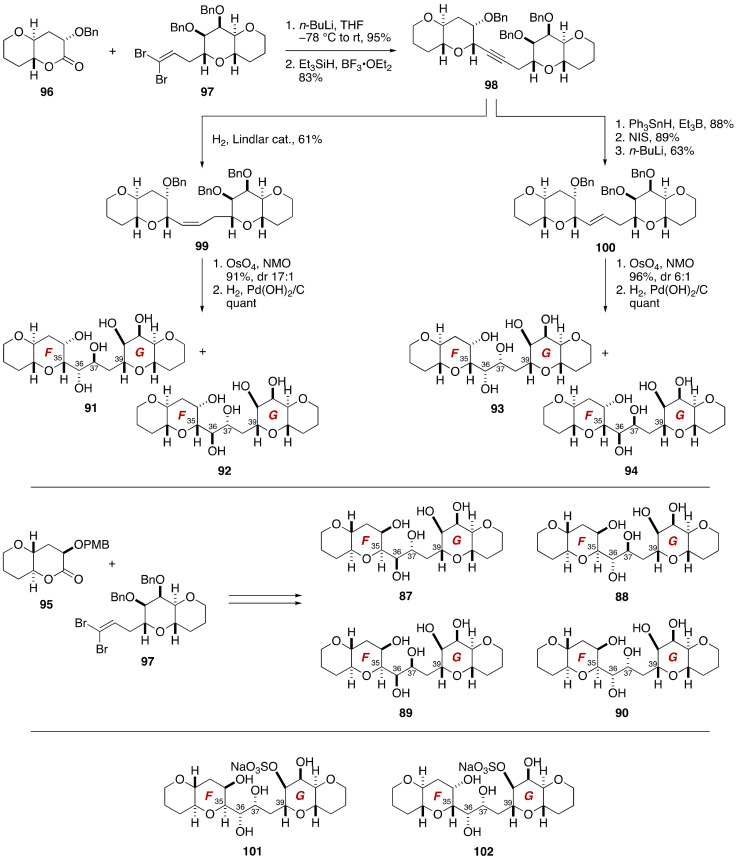
Synthesis of model compounds **87**–**94**, **101**, and **102**.

The synthesis of model compounds **87**–**94** was achieved in a unified manner through a convergent assembly of the EF-ring lactone **95**/**96** and the GH-ring dibromoolefin **97** (Figure 19). These small cyclic ether fragments were available from methyl α-d-galactopyranoside and methyl α-d-mannopyranoside [78,79]. Coupling of **96** and **97** (*n*-BuLi, THF, −78 °C to rt, 95%) followed by reductive etherification gave alkyne **98** (83%). This compound served as a common precursor of four diastereomers **91**–**94**. Semi-reduction of **98** by hydrogenation under the influence of Lindlar catalyst provided *cis*-olefin **99**. Dihydroxylation of **99** delivered a 17:1 mixture of diols. Hydrogenolysis of the benzyl ethers furnished model compounds **91** and **92**. Meanwhile, hydrostannation of **98** under radical conditions followed by Sn–I exchange, and halogen–lithium exchange/proton quench led to *trans*-olefin **100**. Dihydroxylation of **100** gave a 6:1 mixture of diols. Debenzylation afforded model compounds **93** and **94**. In a similar manner, model compounds **87**–**90** were synthesized from the EF-ring lactone **95** and the GH-ring dibromoolefin **97**. Upon examination of the ^1^H and ^13^C NMR data of the eight diastereomers **87**–**94**, diastereomers **90** and **93** were found to be in close match with natural maitotoxin. The Kishi group further synthesized two sulfated model compounds **101** and **102** to identify that the structure **102** represents the relative configuration of the C35–C39 acyclic portion [69].

#### 2.2.3. Stereochemical Assignment of the C63–C68 Acyclic Portion

**Figure 20:**
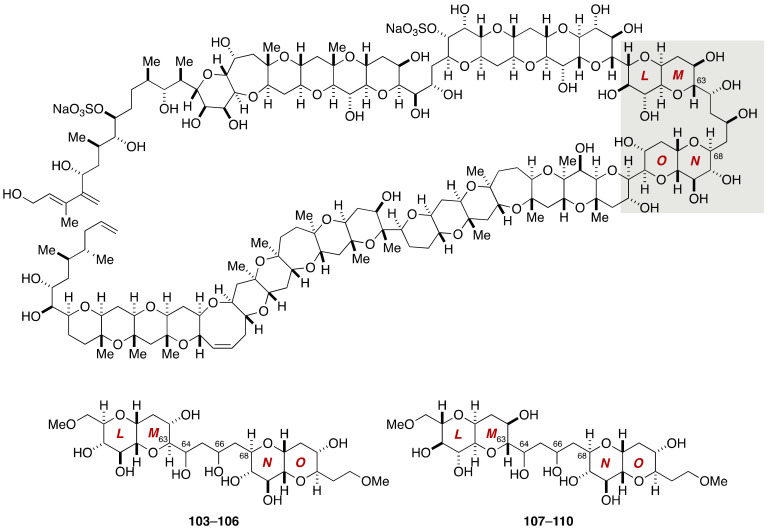
Model compounds **103**–**110** for stereochemical assignment of the C63–C68 acyclic portion.

The Kishi group synthesized all eight possible diastereomers for stereochemical assignment of the C63–C68 acyclic portion containing four stereogenic centers (Figure 20).

**Figure 21:**
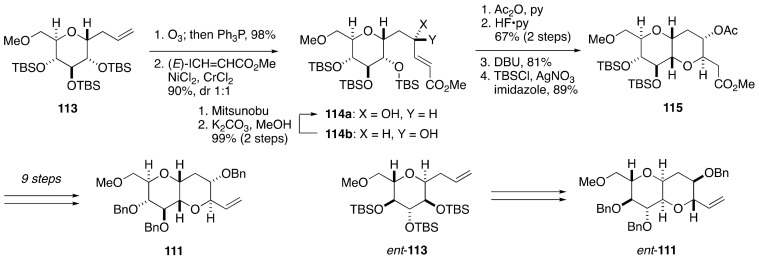
Synthesis of olefin **111**/*ent*-**111**.

**Figure 22:**
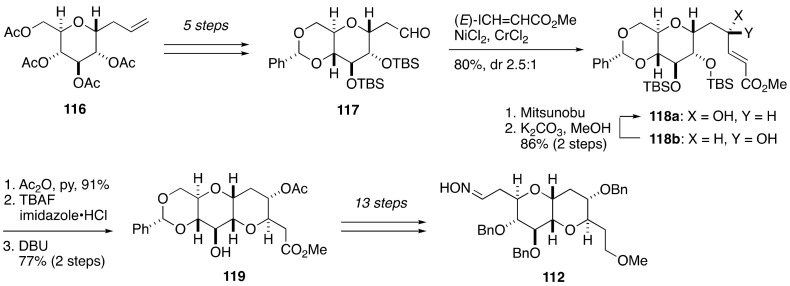
Synthesis of aldoxime **112**.

**Figure 23:**
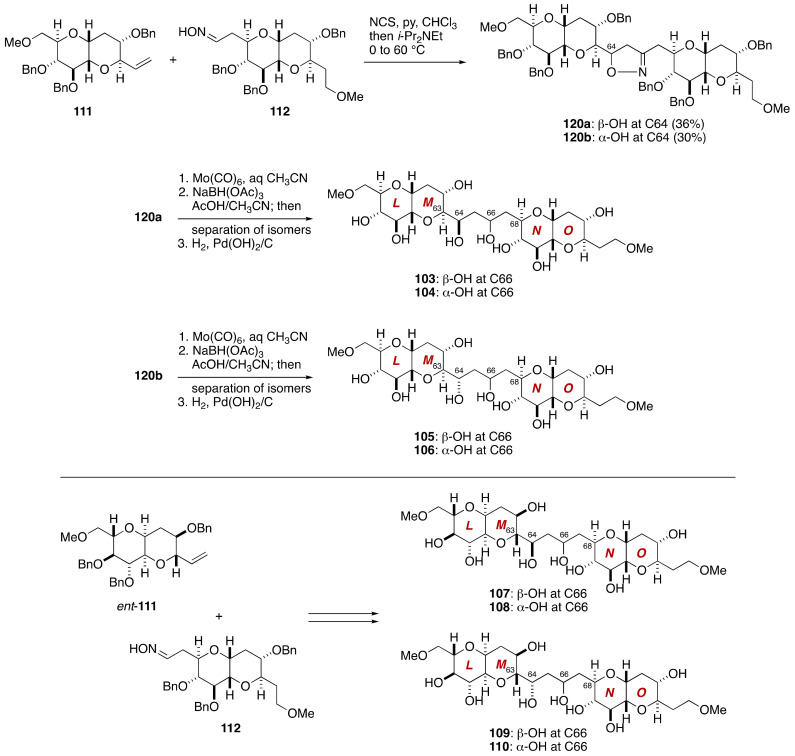
Synthesis of model compounds **103**–**110**.

The synthesis of model compounds **103**–**106** was based on a convergent assembly of olefin **111** and aldoxime **112** (Figure 21, Figure 22 and Figure 23). Olefin **111** was synthesized from *C*-glycoside **113**, the latter being available from 1,6-anhydro-d-glucose in five steps (Figure 21). Ozonolysis of the double bond followed by Nozaki-Hiyama-Kishi coupling with methyl (*E*)-β-iodoacrylate delivered alcohols **114a** and **114b** as an approximately 1:1 mixture of diastereomers, which were separable by column chromatography. The undesired diastereomer could be transformed into the desired one via a Mitsunobu reaction [80]/methanolysis sequence. After acetylation and desilylation, the resultant triol was treated with DBU to induce intramolecular oxa-Michael addition [81,82], giving rise to bicyclic ether **115**, after re-silylation of the resulting two hydroxy groups. A nine-step sequence of manipulations led to olefin **111**. The enantiomer of **111**, i.e., *ent*-**111**, was prepared from 1,6-anhydro-l-glucose in the same manner.

Meanwhile, the synthesis of aldoxime **112** started from *C*-glycoside **116** (Figure 22). After five steps of standard manipulations, the derived aldehyde **117** was reacted with methyl (*E*)-β-iodoacrylate under Nozaki-Hiyama-Kishi conditions to deliver alcohols **118a** and **118b** in 80% yield with 2.5:1 diastereoselectivity. These diastereomers were separated by column chromatography on silica gel, and the undesired minor diastereomer **118b** was converted to the desired **118a** via a Mitsunobu reaction/methanolysis sequence. Acetylation followed by desilylation using buffered TBAF gave a diol, which upon exposure to DBU underwent intramolecular oxa-Michael addition to provide bicyclic ether **119**. This compound was manipulated over 13 steps to afford aldoxime **112**.

Coupling of olefin **111** and aldoxime **112** provided 1,3-dipolar addition product **120a**,**b** in 50–60% yield with 3:2 diastereoselectivity (Figure 23). These diastereomers were separated by silica gel column chromatography. Treatment of the major diastereomer **120a** with Mo(CO)_6_ in aqueous CH_3_CN [83] followed by reduction of the derived β-hydroxy ketone with NaBH(OAc)_3_ in AcOH/CH_3_CN gave 1,3-diols as an approximately 4:1 mixture of diastereomers at C66. These diastereomers were separated by silica gel column chromatography, and individually subjected to debenzylation to afford model compounds **103** and **104**. The minor diastereomer **120b** of the 1,3-dipolar addition product was similarly processed to provide model compounds **105** and **106**. The remainder of model compounds, i.e., **107**–**110**, was synthesized via a coupling of olefin *ent*-**111** and aldoxime **112**.

Among the eight diastereomeric model compounds, the ^1^H and ^13^C NMR spectroscopic data of **105** matched excellently those of the corresponding part of natural maitotoxin [69]. The relative configuration of **105** was secured by derivatization and NMR spectroscopic analysis of late-stage intermediates derived from the 1,3-dipolar adduct **120b**.

#### 2.2.4. Stereochemical Assignment of the C134–C142 Side Chain

**Figure 24:**
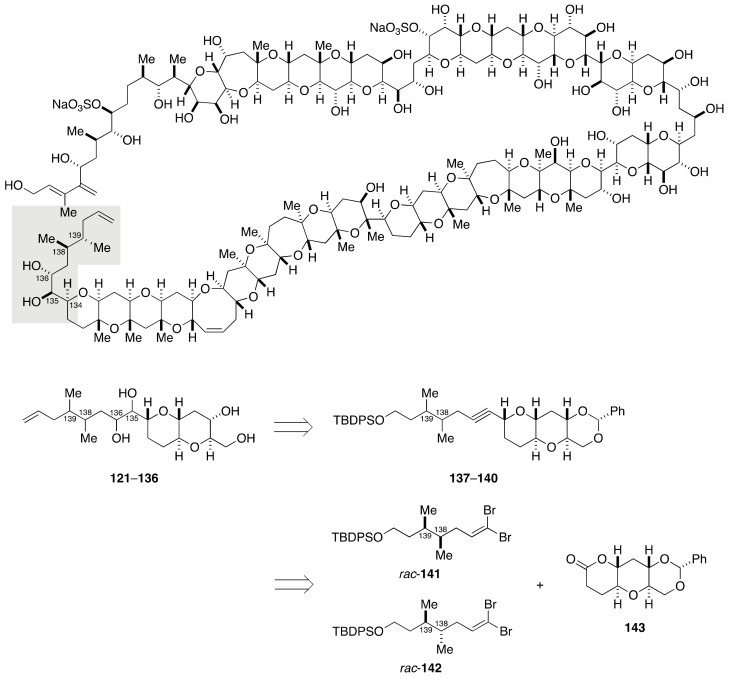
Model compounds **121**–**136** for stereochemical assignment of the C134–C142 side chain.

The relative configurational assignment of the C134–C142 side chain of maitotoxin required the synthesis of 16 diastereomeric model compounds **121**–**136** (Figure 24). The Kishi group synthesized these diastereomers from four diastereomeric alkynes **137**–**140** by means of dihydroxylation of (*E*)- and (*Z*)-olefins. The alkynes **137**–**140** in turn would be obtainable through coupling of two racemic dibromoolefins *rac*-**141**/*rac*-**142** with optically active lactone **143**, followed by separation of diastereomers.

**Figure 25:**
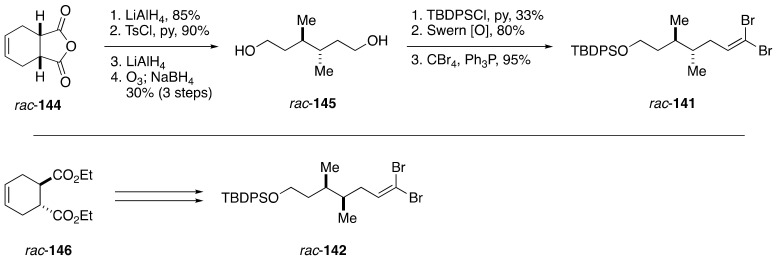
Synthesis of dibromoolefins *rac*-**141**/*rac*-**142**.

The synthesis of dibromoolefin *rac*-**141** started with LiAlH_4_ reduction of Diels-Alder cycloaddition product *rac*-**144**, followed by tosylation/reduction to remove superfluous hydroxy groups, giving *cis*-4,5-dimethylcyclohexene (Figure 25). Subsequent ozonolysis and NaBH_4_-workup delivered diol *rac*-**145**. Monosilylation followed by an oxidation/dibromoolefination sequence provided dibromoolefin *rac*-**141**. In a similar fashion, dibromoolefin *rac*-**142** was prepared from Diels–Alder cycloaddition product *rac*-**146**.

**Figure 26:**
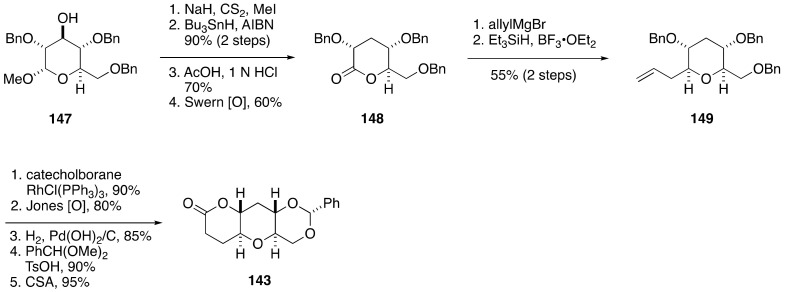
Synthesis of lactone **143**.

The coupling partner **143** was available from methyl α-d-glucopyranoside derivative **147** (Figure 26). Barton–McCombie deoxygenation of the hydroxy group via a xanthate, followed by acidic hydrolysis of the methyl glycoside moiety and Swern oxidation of the derived hemiacetal provided lactone **148**. Addition of allylmagnesium bromide to **148** and reductive etherification of the resultant hemiacetal afforded *C*-glycoside **149**. A five-step sequence of manipulations including hydroboration of the terminal olefin, Jones oxidation, debenzylation, benzylidene acetal protection, and lactonization delivered lactone **143**.

**Figure 27:**
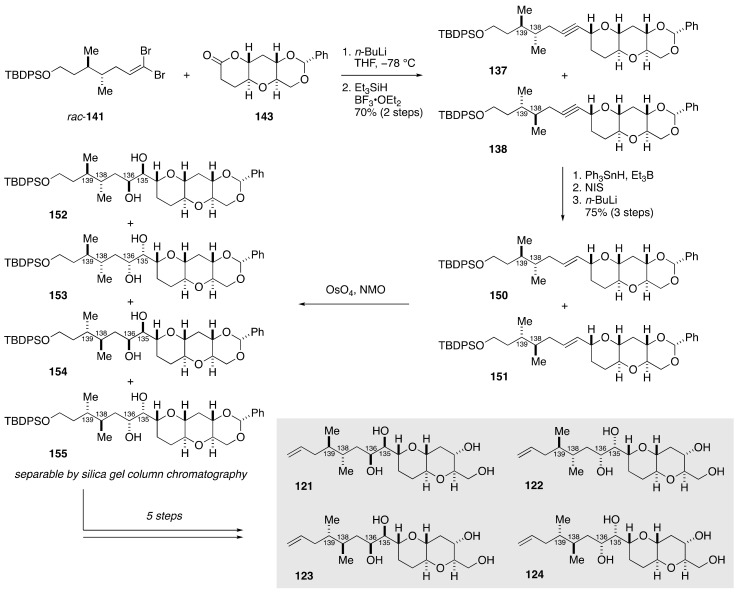
Synthesis of model compounds **121**–**124**.

**Figure 28:**
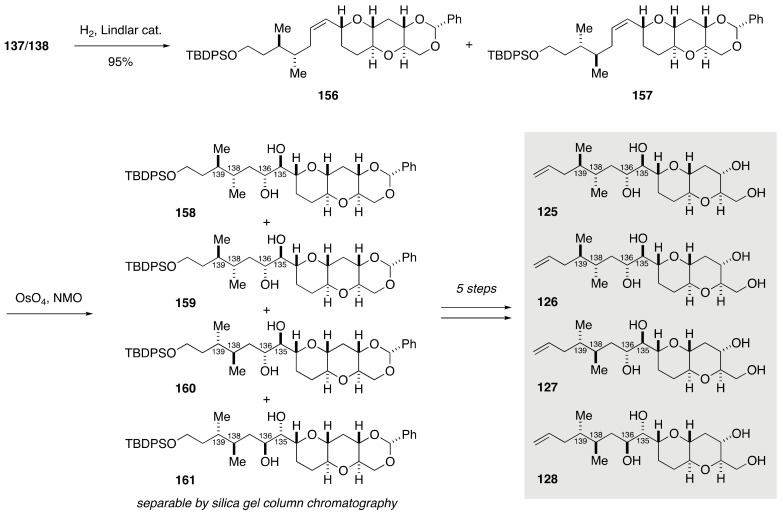
Synthesis of model compounds **125**–**128**.

**Figure 29:**
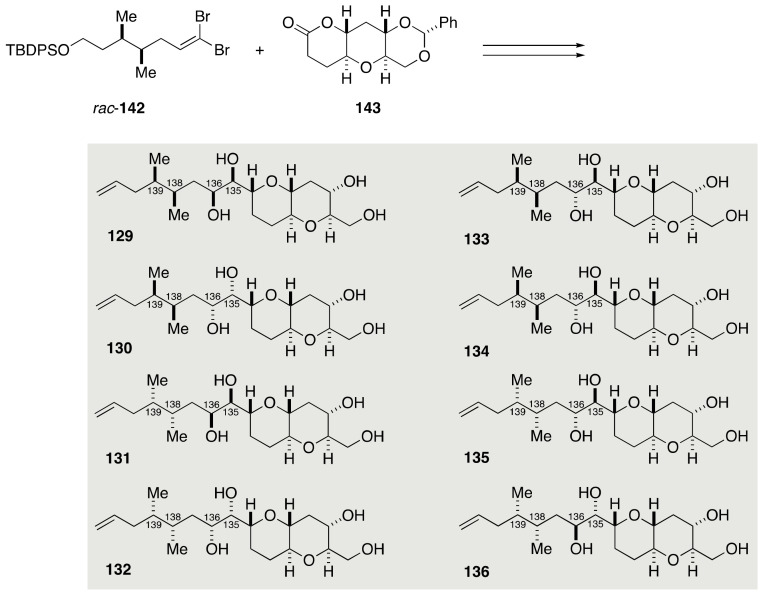
Synthesis of model compounds **129**–**136**.

Coupling of dibromoolefin *rac*-**141** and lactone **143**, followed by reductive etherification of the resultant product, delivered a diastereomeric mixture of alkynes **137** and **138** (Figure 27). Without separation, this mixture was transformed to a mixture of (*E*)-olefins **150**/**151** over three steps. Dihydroxylation of **150**/**151** provided four diastereomeric *threo* diols **152**–**155**, which were separable by silica gel column chromatography. A series of standard manipulations furnished model compounds **121**–**124**. Meanwhile, semi-reduction of **137**/**138** gave a mixture of (*Z*)-olefins **156**/**157**, whose dihydroxylation delivered four diastereomeric *erythro* diols **158**–**161** (Figure 28). These diastereomers were chromatographically separable, and subjected individually to a five-step sequence of manipulations to afford model compounds **125**–**128**. In a similar manner, model compounds **129**–**136** were synthesized from dibromoolefin *rac*-**142** and lactone **143** (Figure 29). The ^1^H and ^13^C NMR spectroscopic data of model compounds **121**–**136** were compared with those of the corresponding portion of natural maitotoxin to show that model compound **121** was the best match. The configuration of **121** was confirmed by its independent synthesis from (*S*)-citronellal as a source of the C139 stereogenic center.

Overall, the Kishi group has successfully established the relative configuration of maitotoxin [69]. Moreover, they proposed the absolute configuration of this natural product on the basis of biosynthetic considerations, which turned out to be same as that determined experimentally by the Tachibana/Yasumoto group [27]. In the event, the Kishi group determined the absolute configuration of maitotoxin independently (see the Note Added in Proof of [69]).

### 2.3. Proposal of the Alternative Configuration of the JK-Ring Juncture by Gallimore and Spencer, and Its Disproof by Confirmation of the Originally Assigned Configuration of the JK-Ring Juncture by the Nicolaou Group

**Figure 30:**
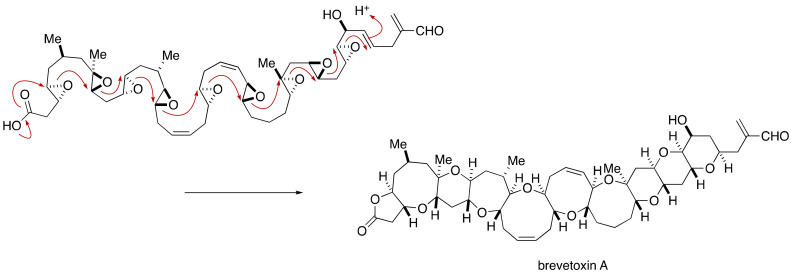
Proposed polyepoxide cyclization cascade for the biosynthesis of brevetoxin A.

The biosynthesis of marine polycyclic ether natural products has not been fully understood. Nakanishi and Shimizu independently proposed a biosynthetic mechanism of brevetoxin A [12,13], in which a single carbon-chain polyene is epoxidized to an octaepoxide precursor and then a cascade of polyepoxide cyclization takes place to “zip up” the polycyclic ether core (Figure 30). The strict *trans*/*syn*/*trans* stereochemical regularity shared among the family of marine polycyclic ether natural products suggests that a common mechanism is operating for their biosynthesis and that a polyene should be epoxidized from the same face of the molecule by a single monooxygenase enzyme.

**Figure 31:**
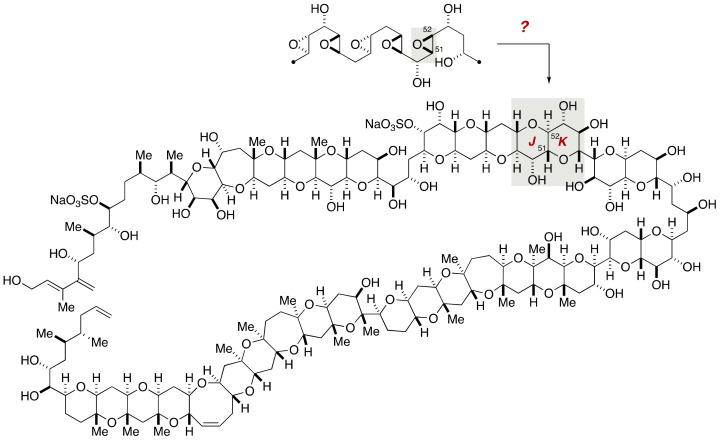
The configuration of the JK-ring juncture of maitotoxin and assumed biosynthetic model.

In 2006, Gallimore and Spencer pointed out that the configuration of the JK-ring juncture (C51/C52) of maitotoxin is an exceptional example that deviates from the common stereochemical regularity of marine polycyclic ethers (Figure 31) [84]. Assuming the polyepoxide cyclization cascade, the JK-ring juncture should arise from an (*S*,*S*)-*trans*-epoxide, whereas the other ring junctures are derived from (*R*,*R*)-*trans*-epoxides. It appears odd that a monooxygenase epoxidizes all but one double bond from the same face of a polyene and another monooxygenase epoxidizes specifically the double bond in question from the opposite face. To make the matter complicated, the configurational assignment of the JK-ring juncture had been noted as being highly challenging due to heavy overlap of NMR signals [85]. Accordingly, Gallimore and Spencer proposed that the configuration of the JK-ring juncture should be opposite to that determined in previous works.

In 2007, Nicolaou and Frederick suggested on the basis of DFT chemical shift calculations that the originally proposed configuration of the JK-ring juncture is likely correct, and proposed an alternative biosynthetic route that does not violate the hypothesis of Gallimore and Spencer [86]. Nicolaou and coworkers reported the synthesis of a GHIJK ring model compound in 2007 [87] and the GHIJKLMNO ring domain in 2008 [88] to provide a strong evidence that supports the originally assigned configuration.

**Figure 32:**
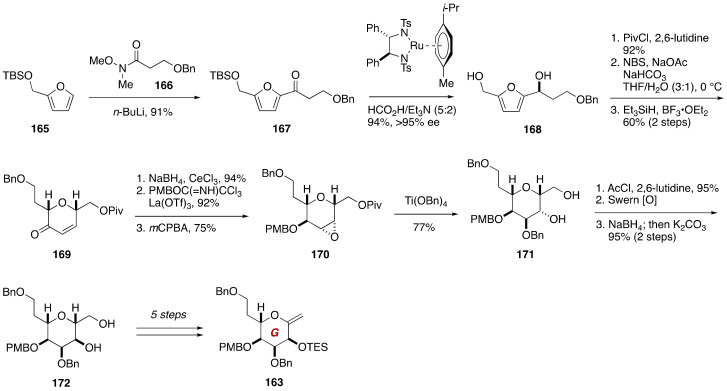
Synthesis of the G-ring exocyclic enol ether **163**.

**Figure 33:**
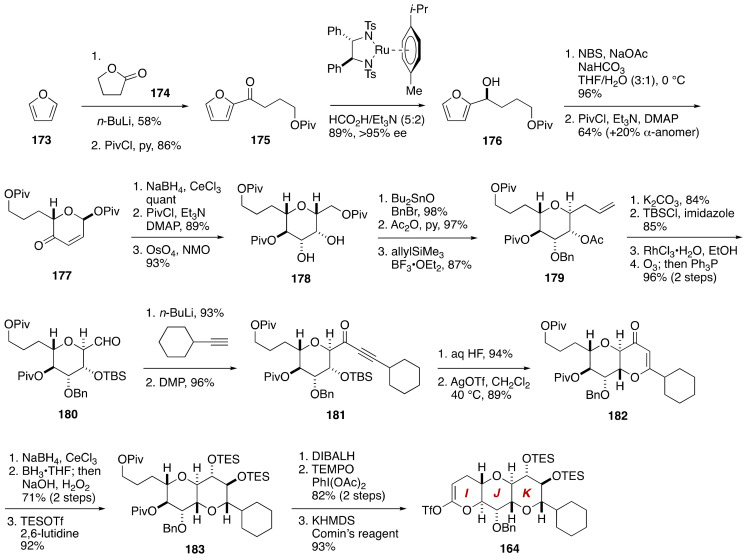
Synthesis of the IJK-ring enol triflate **164**.

**Figure 34:**
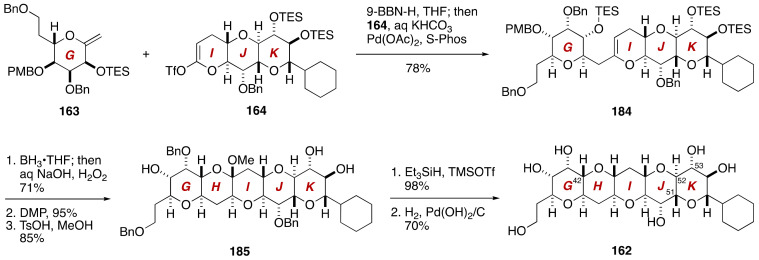
Synthesis of the GHIJK-ring model compound **162**.

The Nicolaou synthesis of the GHIJK ring model compound **162** was achieved based on a Suzuki–Miyaura coupling [89,90,91,92] of the G-ring exocyclic enol ether **163** and the IJK-ring enol triflate **164** (Figure 32, Figure 33 and Figure 34). The G-ring enol ether **163** was prepared from furan **165** (Figure 32). Lithiation of **165** with *n*-BuLi and in situ trapping of the generated alkenyl lithium species with Weinreb amide **166** gave ketone **167**. Noyori asymmetric hydrogenation [93] of **167** led to alcohol **168** (>95% ee). After selective protection of the primary hydroxy group, Achmatowicz rearrangement (NBS, NaOAc, NaHCO_3_, THF/H_2_O (3:1), 0 °C) [94] and subsequent silane reduction of the resultant hemiacetal delivered α,β-unsaturated ketone **169**. Stereoselective Luche reduction,[95] protection of the derived alcohol, and epoxidation with *m*CPBA gave epoxide **170**. Regioselective epoxide opening of **170** with Ti(OBn)_4_ [96] accompanied cleavage of the pivaloate to provide diol **171**. A three-step sequence of manipulations including a sequence of oxidation/reduction to invert the configuration of the secondary alcohol delivered diol **172** with correct configuration for the G-ring. This intermediate was converted to the G-ring exocyclic enol ether **163** in five steps.

The IJK-ring enol triflate **164** was prepared from furan (**173**) by adopting Achmatowicz rearrangement chemistry (Figure 33). Lithiation/acylation followed by pivaloylation of the resultant alcohol gave pivaloate **175**, which was reduced under the Noyori conditions to deliver alcohol **176** (>95% ee). Achmatowicz rearrangement of **176** and pivaloylation of the derived hemiacetal led to α,β-unsaturated ketone **177**.

Luche reduction of **177** followed by acylation, and subsequent dihydroxylation provided diol **178**. After a two-stage protecting group manipulations to differentiate the hydroxy groups, allylation with allyltrimethylsilane/BF_3_•OEt_2_ afforded olefin **179**. After replacement of the acetyl group with a TBS group, migration of the double bond followed by ozonolysis gave aldehyde **180**, which was alkynylated with lithiated cyclohexylacetylene and then oxidized to deliver α,β-unsaturated ketone **181**. Desilylation and subsequent activation of the alkyne with a catalytic amount of AgOTf (CH_2_Cl_2_, 40 °C) provided dihydropyranone **182**. A three-step functionalization of the dihydropyranone ring, including Luche reduction, hydroboration, and bis-silylation, led to bis-silyl ether **183**. After reducing the pivaloates, oxidative lactonization of the derived diol, followed by enolization/triflation, gave rise to the IJK-ring enol triflate **164**.

Hydroboration of the G-ring exocyclic enol ether **163** with 9-BBN-H and coupling of the resultant alkylborane with the IJK-ring enol triflate **164** under the influence of the Pd(OAc)_2_/S-Phos [97] catalyst and KHCO_3_ as a base (THF/H_2_O, room temperature) afforded coupling product **184** in 78% yield (Figure 34). Hydroboration with BH_3_•THF, Dess-Martin oxidation,[98] and subsequent desilylation/methyl acetalization delivered methyl acetal **185**, which was reduced with Et_3_SiH/TMSOTf and then debenzylated to furnish the GHIJK ring model compound **162**. The ^13^C NMR spectroscopic data of the C42–C53 moiety of **162** were in excellent agreement with those of the corresponding portion of natural maitotoxin [87].

## 3. Brevenal

**Figure 35:**
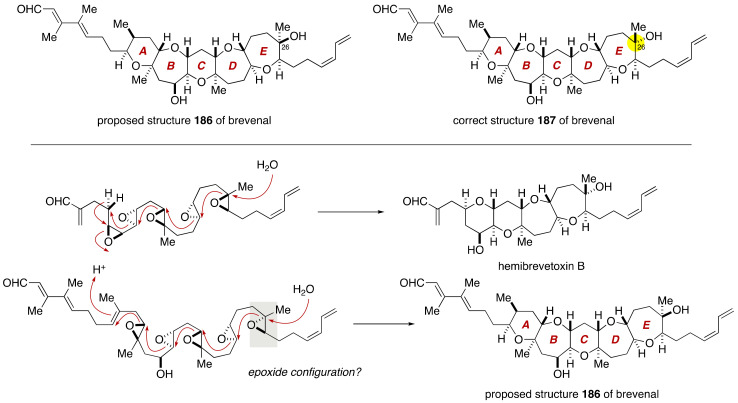
Proposed and correct structures of brevenal, and biosynthetic consideration.

Bourdelais, Baden and coworkers isolated brevenal from a laboratory culture of the Florida red tide-forming dinoflagellate *Karenia brevis* [99]. This natural product competitively inhibits the binding of tritiated dihydrobrevetoxin B ([^3^H]PbTx-3) to site 5 of voltage-gated sodium ion channels in a dose-dependent fashion without neurotoxicity and acts as a naturally occurring brevetoxin antagonist in vivo. Brevenal is also a potent inhibitor of catecholamine secretion induced by ciguatoxin without affecting other secretagogue activities, including nicotine- or barium-induced catecholamine secretion [100,101]. Ciguatoxin is the principal causative toxin of ciguatera sea food poisoning and exhibits potent neurotoxicity by binding to site 5 of voltage-gated sodium ion channels. Accordingly, it has been suggested that brevenal could be potentially useful for the treatment of ciguatera. Moreover, brevenal improves tracheal mucus clearance activity in an animal model of asthma, suggesting that it might be useful as a candidate in the drug development toward cystic fibrosis therapy [102]. The Baden group determined the gross structure of brevenal through extensive 2D-NMR analyses, and assigned the relative configuration on the basis of NOESY correlations. However, the absolute configuration of this natural product remained unassigned. The structure of brevenal is reminiscent of that of hemibrevetoxin B, a tetracyclic ether metabolite of *K. brevis* [103]. According to the Nakanishi hypothesis [10], these compounds should be biosynthetically generated from the respective polyepoxides via a cascade ring opening, as shown in Figure 35. The configuration of the C26 stereogenic center of the proposed structure **186** of brevenal appeared unusual on the basis of the biosynthetic consideration. Actually, the Sasaki group later revised the configuration of the C26 stereogenic center through total synthesis [104,105,106,107].

**Figure 36:**
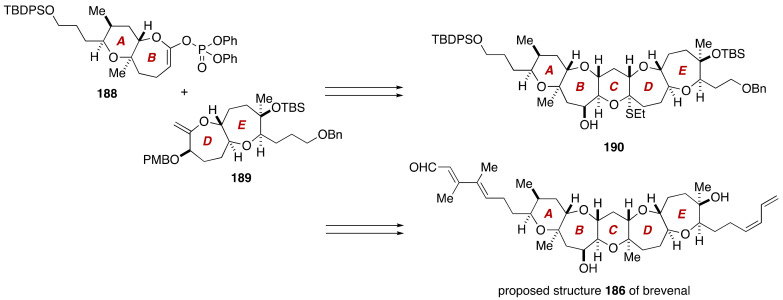
Synthesis plan toward the proposed structure **186** of brevenal.

The first total synthesis of the proposed and correct structures of brevenal was accomplished by Sasaki and coworkers [104,105,106,107]. As shown in Figure 36, the Sasaki group built up the pentacyclic polyether core of **186** through a Suzuki–Miyaura coupling [89,90,91,92] of the AB-ring enol phosphate **188** and an alkylborane derived from the DE-ring exocyclic enol ether **189**, followed by a stereoselective methylation of thioacetal **190**. The unsaturated side chains at both ends of the polycyclic ether skeleton were introduced by means of a Stille reaction [108] and a Wittig olefination.

**Figure 37:**
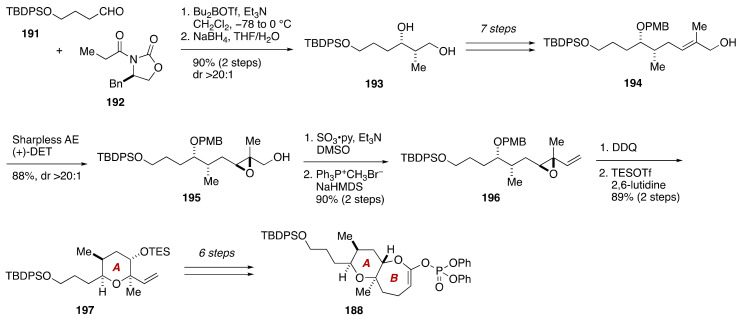
Synthesis of the AB-ring enol phosphate **188**.

The synthesis of the AB-ring enol phosphate **188** started with Evans *syn*-aldol reaction [109] of aldehyde **191** and *N*-propionyl (*R*)-4-benzyl-2-oxazolidinone (**192**), and subsequent reductive removal of the chiral auxiliary [110] provided 1,3-diol **193** (Figure 37). The diol **193** was elaborated to allylic alcohol **194** in seven steps. Sharpless asymmetric epoxidation of **194** using (+)-DET as a chiral ligand delivered epoxy alcohol **195**. After oxidation and Wittig methylenation, the resultant vinyl epoxide **196** was exposed to DDQ to induce cleavage of the PMB ether and concomitant 6-*endo* cyclization, leading to tetrahydropyran **197**, after protection of the resultant alcohol as its TES ether (89%, two steps). Additional six steps, including Yamaguchi lactonization [111] to construct the seven-membered ring, delivered the AB-ring enol phosphate **188**.

**Figure 38:**
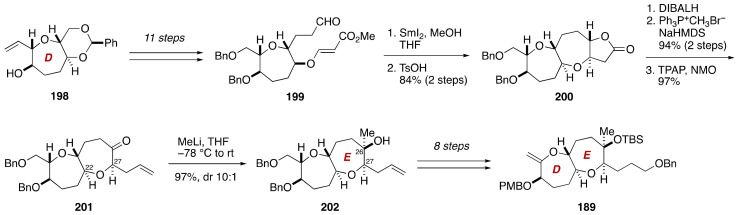
Synthesis of the DE-ring exocyclic enol ether **189**.

The synthesis of the DE-ring exocyclic enol ether **189** started from known alcohol **198** [112] that corresponds to the D-ring (Figure 38). The alcohol **198** was manipulated over 11 steps to aldehyde **199**. Exposure of **199** to SmI_2_ (MeOH, THF, room temperature) under Nakata conditions [113,114] resulted in reductive cyclization to form the E-ring seven-membered ether, affording lactone **200** after acid treatment. The lactone **200** was transformed to ketone **201** via a three-step sequence of manipulations, at which point the relative configuration of the C22/C27 stereogenic centers was confirmed by an NOE. Introduction of the C26 methyl group was most reliably achieved by treatment of **201** with MeLi (THF, −78 °C to room temperature), giving rise to tertiary alcohol **202** in 97% yield with 10:1 diastereoselectivity. The configuration of the newly generated C26 stereogenic center was determined by observing an NOE enhancement between the C26 methyl group and the C27 oxymethine proton. Subsequent eight-step manipulations afforded the DE-ring exocyclic enol ether **189**.

**Figure 39:**
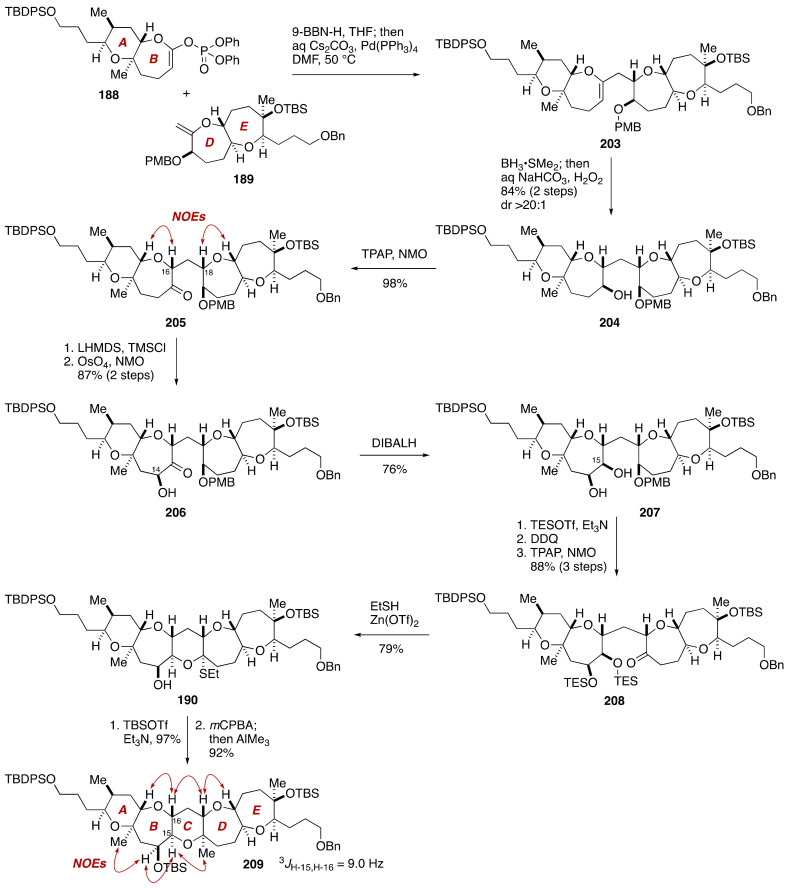
Synthesis of the pentacyclic polyether core **209**. Double-ended arrows denote NOEs.

Suzuki–Miyaura coupling of an alkylborane, derived from the DE-ring exocyclic enol ether **189** via hydroboration with 9-BBN-H, and the AB-ring enol phosphate **188** proceeded uneventfully under the influence of aqueous Cs_2_CO_3_ and Pd(PPh_3_)_4_ in THF/DMF at 50 °C to deliver endocyclic enol ether **203** (Figure 39). Hydroboration using BH_3_•SMe_2_ and alkaline oxidative workup provided alcohol **204** in 84% yield (two steps) as a single diastereomer (dr > 20:1). Oxidation of **204** under Ley’s conditions [115] led to ketone **205** in 98% yield, at which point the configuration of the C16 and C18 stereogenic centers was confirmed by NOE correlations as shown. The C14 hydroxy group was introduced at this stage via a diastereoselecive dihydroxylation of an enol silyl ether derived from **205**, giving rise to α-hydroxy ketone **206** (87%, two steps) with greater than 20:1 diastereoselectivity. Stereoselective reduction of **206** with DIBALH (THF, −78 °C) delivered *cis*-diol **207** in 76% yield, along with its C15 epimer (not shown) in 7% yield (starting material was recovered in 12% yield). The configuration of the C14 and C15 stereogenic centers was confirmed by NOE correlations observed for the corresponding cyclopentylidene derivative. After a three-step sequence of manipulations, the derived ketone **208** was exposed to EtSH/Zn(OTf)_2_ [116] to promote cleavage of the TES ethers and spontaneous thioacetal formation, affording thioacetal **190** in 79% yield. The construction of the C-ring was completed by silylation of the hydroxy group of **190** and subsequent *m*CPBA oxidation and in situ methylation with AlMe_3_ (CH_2_Cl_2_, −78 to 0 °C) [117] to furnish the pentacyclic ether **209** in 92% yield as a single stereoisomer (dr > 20:1). At this point, the configuration of **209** was further corroborated by NOE experiments and a ^3^*J*_H,H_ value.

**Figure 40:**
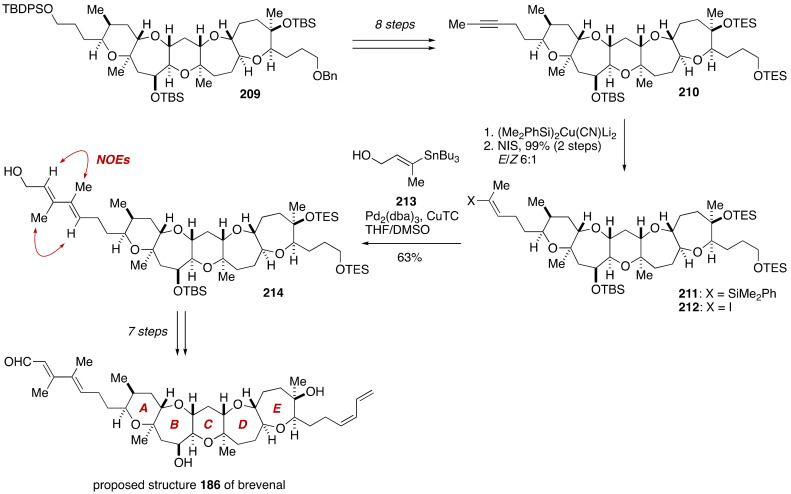
Total synthesis of the proposed structure **186**. Double-ended arrows denote NOEs.

Finally, the side chains at both ends of the pentacyclic ether framework were attached to complete the total synthesis (Figure 40). Thus, the pentacyclic ether **209** was converted to alkyne **210** in eight steps. Stannylcupuration of **210** with (Me_2_PhSi)_2_Cu(CN)Li_2_ (THF, −78 to 0 °C) [118] delivered vinylsilane **211** with 9:1 regioselectivity, and ensuing iododesilylation with NIS [119] provided vinyl iodide **212** in 99% yield with *E*/*Z* 6:1 selectivity. Stille reaction of **212** with vinylstannane **213** under the influence of Pd_2_(dba)_3_/Ph_3_As and CuTC [120] in DMSO/THF at room temperature afforded conjugated diene **214** in 63% yield after removal of minor isomers by flash column chromatography using silica gel. The configuration of the conjugated diene moiety was confirmed by NOE experiments. Subsequent seven-step manipulations, including a Wittig olefination/selenoxide elimination to install the right-hand side chain according to the procedure of Nicolaou and coworkers [121], furnished the proposed structure **186** of brevenal [104].

**Figure 41:**
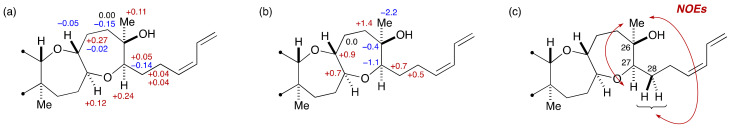
Chemical shift deviation analysis and important NOE contacts observed for synthetic **186**. (**a**) ^1^H NMR chemical shift deviations. Δδ = δ(natural) − δ(synthetic **186**). (**b**) ^13^C NMR chemical shift deviations. Δδ= δ(natural) − δ(synthetic **186**). (**c**) Diagnostic NOEs observed for synthetic **186**.

However, the ^1^H and ^13^C NMR spectra of synthetic **186** do not match those of the authentic material. The COSY, HSQC, and HMBC correlations of synthetic **186** reproduced those of the natural product, indicating that the gross structure of **186** was correctly assigned. Upon chemical shift deviation analysis, significant inconsistencies were found in around the C26 tertiary alcohol (Figure 41). Moreover, intense NOESY correlations were observed between C26 methyl/C27 methine and C26 methyl/C28 methylene protons of synthetic **186**, whereas no such NOESY correlations were observed for natural brevenal. Thus, it was suggested that the configuration of the C26 stereogenic center might have been erroneously assigned. Importantly, this assumption was in accordance with the biosynthetic consideration described above.

**Figure 42:**
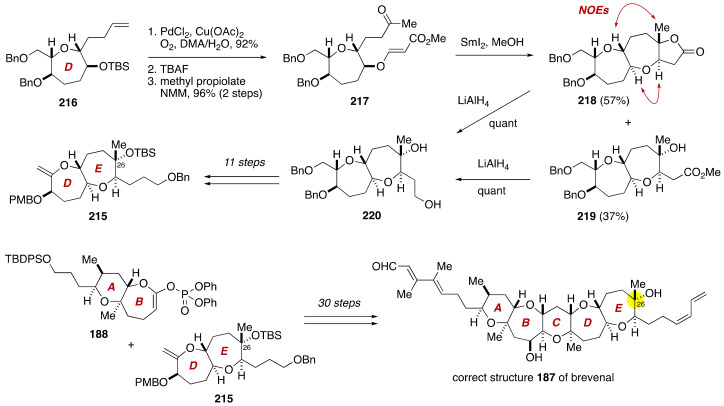
Total synthesis of the correct structure **187**. Double-ended arrows denote NOEs.

Eventually, the Sasaki group embarked on the total synthesis of the revised structure **187** of brevenal, as shown in Figure 42. The DE-ring exocyclic enol ether **215** (26-*epi*-**189**) was synthesized from olefin **216**, the latter being available from **198**. A three-step sequence of manipulations gave ketone **217**. SmI_2_-mediated reductive cyclization of **217** under Nakata conditions provided a mixture of lactone **218** (57%) and hydroxy ester **219** (37%), which were separable by flash column chromatography using silica gel. These products were individually reduced with LiAlH_4_ to give diol **220**. The configuration of the newly formed E-ring was confirmed by NOE experiments on lactone **218** as shown. Subsequent 11-step manipulations afforded the DE-ring exocyclic enol ether **215**. Assembly of the pentacyclic ether skeleton and subsequent introduction of the unsaturated side chains were achieved in the same manner as those described for the proposed structure **186** to complete the total synthesis of the revised structure **187**. As anticipated, the ^1^H and ^13^C NMR spectra of synthetic **187** were fully consistent with those of the natural product. Moreover, the specific rotation value of synthetic **187** ${\left[\alpha \right]}_{D}^{27}$−33.5 (*c* 0.27, benzene) was in close accordance with that of natural brevenal ${\left[\alpha \right]}_{D}^{27}$−32.3 (*c* 0.27, benzene). From these results, the absolute configuration of brevenal was unequivocally established as shown in the structure **187** [105].

## 4. Gambieric Acids

Nagai, Yasumoto and coworkers isolated gambieric acid A and its congeners from a cultured medium of the ciguatera causative dinoflagellate *Gambierdiscus toxicus* as highly potent antifungal substances [18,19]. The structure of gambieric acids consists of a non-acyclic polyether skeleton arranged with an isolated tetrahydrofuran ring. Although the structural characteristics of gambieric acids are similar to those of polycyclic ether neurotoxins produced by *G. toxicus*, gambieric acid A shows no toxicity in mice at a dose of 1 mg/kg (i.p.), and only weakly inhibits binding of tritiated dihydrobrevetoxin ([^3^H]PbTx-3) to site 5 of voltage-gated sodium ion channels [122]. More interestingly, gambieric acid A exhibits potent antifungal activity against *Aspergillus niger* (10 ng/disk, >2000-fold potent than amphotericin B), and it is also suggested to be an endogenous growth regulator of *G. toxicus* [123].

**Figure 43:**
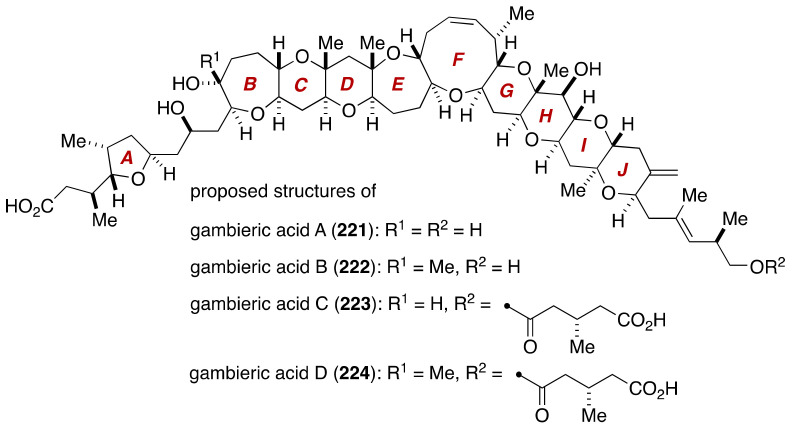
Proposed structures of gambieric acids.

The gross structure and relative configuration of the polycyclic ether skeleton of gambieric acids were determined on the basis of extensive 2D-NMR analyses. The complete configurational assignment of gambieric acid B (structure **222**, Figure 43) was later reported by Satake, Yasumoto and coworkers [20], which entailed degradation experiments, application of chiral anisotropic reagents, chiral HPLC analysis, and conformational analysis based on *J* values and NOE and HMBC correlations. The structures of other members of gambieric acids were assigned accordingly, as shown in Figure 43.

**Figure 44:**
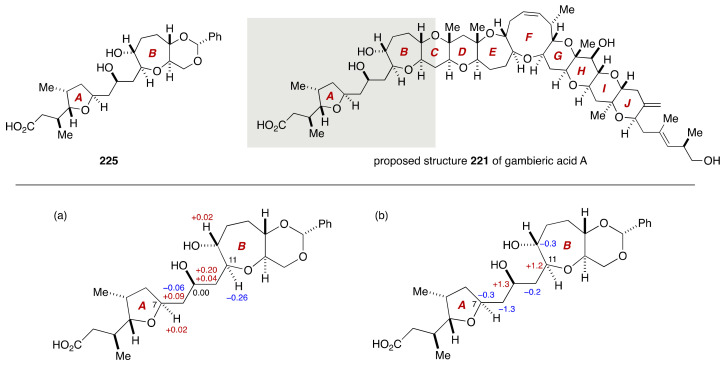
Chemical shift deviations of A/B-ring model **225** of gambieric acid A. (**a**) ^1^H NMR chemical shift deviations. Δδ = δ(natural) − δ(synthetic **225**). (**b**) ^13^C NMR chemical shift deviations. Δδ = δ(natural) − δ(synthetic **225**).

**Figure 45:**
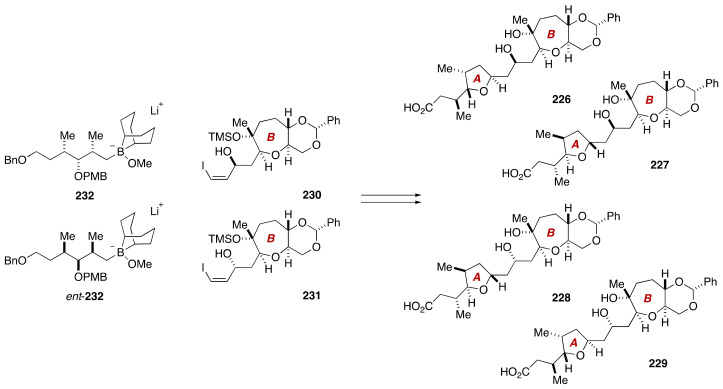
Synthesis plan toward four candidate diastereomers **226**–**229**.

During the course of synthetic studies toward gambieric acids, Fuwa, Sasaki and coworkers found that the ^1^H and ^13^C NMR chemical shifts of an A/B-ring model compound **225** did not match those of the corresponding moiety of natural gambieric acid A (Figure 44) [124,125,126]. Specifically, the C8–C11 moiety of **225** showed significantly deviated chemical shift values, bringing the relative configuration of C7/C9 and C9/C11 into question. Because the C7, C9, and C11 stereogenic centers were configurationally correlated with each other on the basis of conformational analysis on natural gambieric acid B, Fuwa et al. embarked on the synthesis of its four diastereomeric model compounds **226**–**229** through a Suzuki–Miyaura coupling of iodoolefins **230**/**231** with alkylborates **232**/*ent*-**232**, and a diastereoselective bromoetherification to craft the isolated A-ring tetrahydrofuran (Figure 45). The modular synthetic approach was crucial for efficient and stereocontrolled synthesis of all the requisite diastereomers.

**Figure 46:**
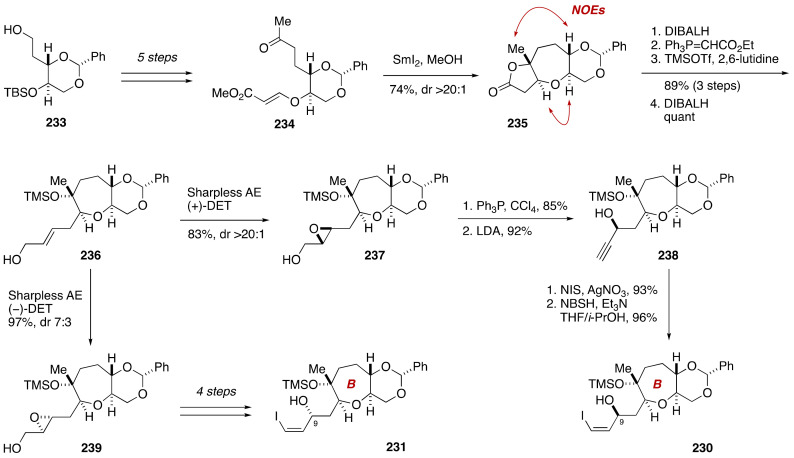
Synthesis of iodoolefins **230**/**231**. Double-ended arrows denote NOEs.

The synthesis of iodoolefins **230**/**231** started with known alcohol **233** [127] (Figure 46). A five-step sequence of standard manipulations led to methyl ketone **234**, which was exposed to SmI_2_ under Nakata conditions to deliver lactone **235** in 74% yield as a single diastereomer (dr > 20:1). The configuration of **235** was confirmed by NOE enhancements as shown. After a four-step sequence of manipulations, Sharpless asymmetric epoxidation of the derived allylic alcohol **236** using (+)-DET as a chiral ligand provided epoxy alcohol **237** in 83% yield with greater than 20:1 diastereoselectivity. Chlorination of **237** followed by treatment with excess LDA [128] gave propargylic alcohol **238**. Iodination of the terminal alkyne and diimide reduction [129] afforded iodoolefin **230**. The configuration of the C9 stereogenic center created through the Sharpless asymmetric epoxidation was confirmed by a modified Mosher analysis [130] on a hydrogenated derivative of **238**. The C9 epimer of **230**, i.e., **231**, was easily available from allylic alcohol **236** by Sharpless asymmetric epoxidation using (−)-DET as a chiral ligand and following the same four-step sequence as described for **230**.

**Figure 47:**
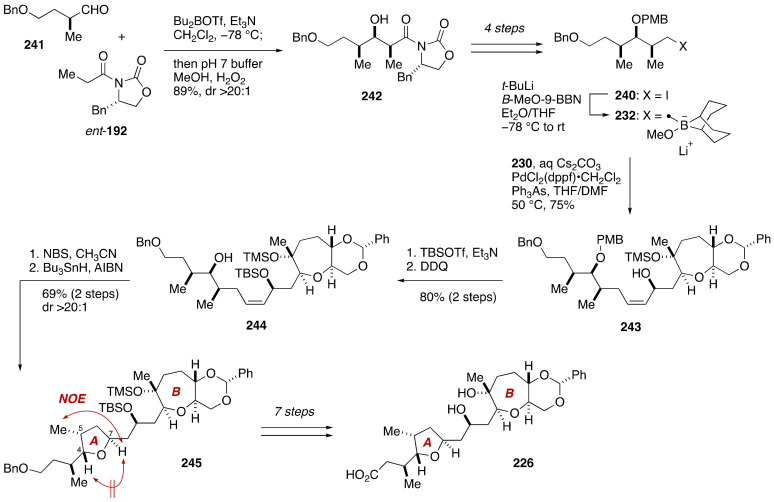
Synthesis of model compound **226**. Double-ended arrows denote NOEs.

The precursor of alkylborate **232**, i.e., iodide **240**, was prepared from known aldehyde **241** [131] over five steps including Evans *syn*-aldol reaction (Figure 47). According to the Marshall procedure [132], iodide **240** was lithiated with *t*-BuLi and trapped with *B*-MeO-9-BBN to generate alkylborate **232**. Without isolation, **232** was coupled with iodoolefin **230** under the influence of aqueous Cs_2_CO_3_ and PdCl_2_(dppf)•CH_2_Cl_2_/Ph_3_As in THF/DMF at 50 °C to deliver allylic alcohol **243** in 75% yield. After two-step protecting group manipulations, diastereoselective bromoetherification of the resultant alcohol **244** (NBS, CH_3_CN, room temperature) and subsequent tin hydride reduction (Bu_3_SnH, AIBN, toluene, 110 °C) afforded 2,5-*trans*-substituted tetrahydrofuran **245** in 69% yield (two steps) as a single diastereomer (dr > 20:1). The configuration of the C4, C5, and C7 stereogenic centers was confirmed by NOE experiments as shown. This compound was elaborated to model compound **226** over seven steps of standard manipulations. Additional three diastereomers **227**–**229** were synthesized from **230**/**231** and **240**/*ent*-**240** in much the same way as described for **226**.

**Figure 48:**
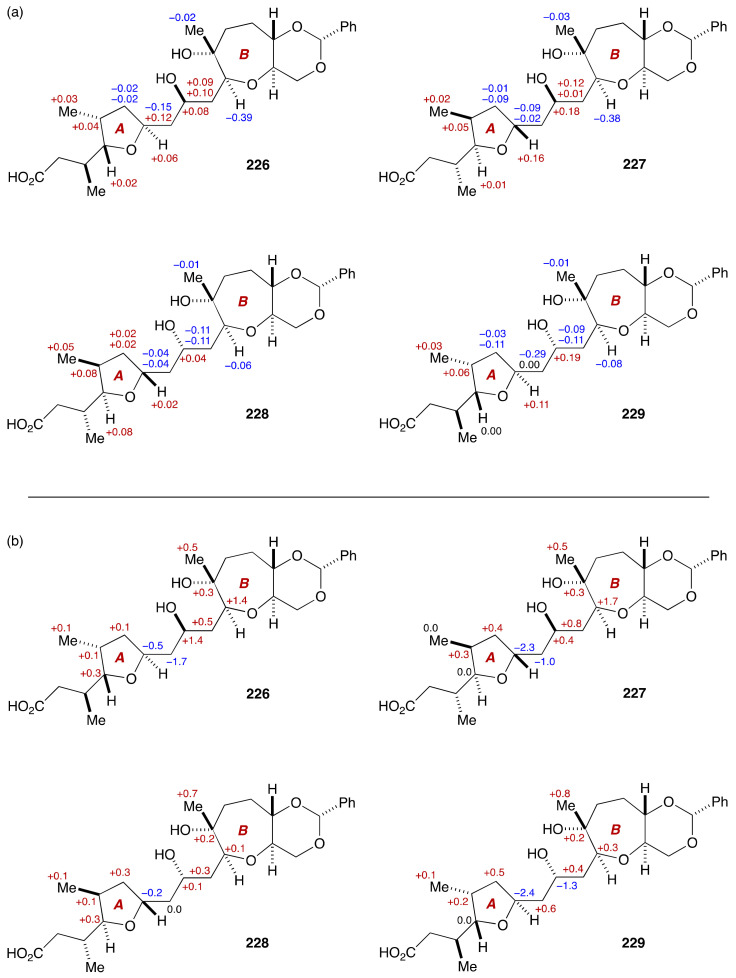
Chemical shift deviation analysis on model compounds **226**–**229**. (**a**) ^1^H NMR chemical shift deviations. Δδ = δ(natural) – δ(synthetic). (**b**) ^13^C NMR chemical shift deviations. Δδ = δ(natural) – δ(synthetic).

**Figure 49:**
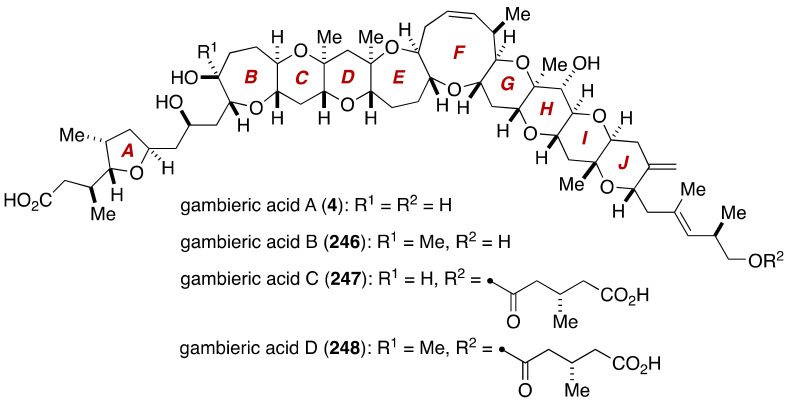
Correct structures of gambieric acids.

Chemical shift deviation analysis of four diastereomeric model compounds **226**–**229** compared with natural gambieric acid B clearly indicated that the ^1^H and ^13^C NMR chemical shifts of **228** were in close agreement with those of the corresponding moiety of the natural product (Figure 48). Moreover, the conformational analysis of **228** on the basis of *J* values and NOESY and HMBC correlations in pyridine-*d*_5_ suggested that the conformation of **228** reproduced faithfully that of the A/B-ring moiety of natural gambieric acid B. Because the configuration of the C9 stereogenic center of **228** is opposite to that of the natural product, it was concluded that the C9/C11 relative configuration had been misassigned in the proposed structure **222**. This resulted in a configurational reassignment of all the stereogenic centers embedded in the nonacyclic polyether skeleton, as shown in Figure 49. This conclusion was further supported by the synthesis/NMR analysis of an A/B-ring model compound of gambieric acid A and an A/BC-ring model compound of gambieric acid B [126]. Moreover, the revised structure of gambieric acid A was ultimately established through the first total synthesis by Fuwa, Sasaki and coworkers [133,134].

## 5. Conclusions

This review summarized the configurational assignment of marine polycyclic ether natural products, maitotoxin, brevenal, and gambieric acids. The maitotoxin case is an illustrative example which demonstrates the power of synthesis-driven configurational assignment of stereochemically complex natural products. At the same time, this case indicates that detailed conformational analysis by means of advanced NMR spectroscopic techniques may be helpful in narrowing down the number of possible diastereomers to be synthesized. The gambieric acid case underlines the significance of the modularity [135] of synthetic planning, which enables expedient access to a set of requisite stereoisomers for determination of the relative configuration in question. These are important points that should be carefully considered in order to save time, costs, and effort in synthesis.

Remarkable advances in NMR spectroscopy in recent decades enable the determination of the gross structure and also the relative configuration of conformationally rigid skeleton of super-carbon-chain polycyclic ethers. Nonetheless, configurational assignment of acyclic portions and remotely isolated stereogenic centers is still a daunting task. The JBCA method is effective for the configurational assignment of non-biased acyclic systems, but its application to more or less biased acyclic systems appears to need special care [136,137,138]. Genome sequence information of the producer organisms may be useful for assigning the configuration of natural products on the basis of biosynthetic predictions, although the biosynthetic genes are difficult to obtain from marine dinoflagellates. Molecular mechanics/DFT calculations may help in assigning those difficult stereogenic centers through the prediction of chemical shift values [139,140]. A recent study on the structure elucidation of gambierone, a novel polycyclic ether metabolite from the dinoflagellate *Gambierdiscus belizeanus*, took advantage of NMR chemical shift calculations at the B3LYP/6-31G(d)//B3LYP/STO-3G level of theory, and assigned, on the basis of DP4 probability analysis [141], the relative configuration of three stereogenic centers within acyclic portions [142]. It should be emphasized, however, that the configurational assignment made by means of computational and/or bioinformatic approaches still needs to be confirmed through synthesis/NMR analysis of suitably designed model compounds and finally be established in an unambiguous manner by total synthesis. Significant challenges remaining in this area are the complete stereochemical assignments of extremely large polycyclic ethers, brevisulcenal-F [143] and prymnesins [144,145].

## Data Availability

Not applicable.
